# Preparation of Antiproliferative Terpene-Alkaloid Hybrids by Free Radical-Mediated Modification of *ent*-Kauranic Derivatives

**DOI:** 10.3390/molecules26154549

**Published:** 2021-07-28

**Authors:** Elena Pruteanu, Vladilena Gîrbu, Nicon Ungur, Leentje Persoons, Dirk Daelemans, Philippe Renaud, Veaceslav Kulcițki

**Affiliations:** 1 Institute of Chemistry (MECC), Str. Academiei, 3, MD-2028 Chișinău, Moldova; elena.pruteanu@unibe.ch (E.P.); vgirbu1@gmail.com (V.G.); nicon.ungur@gmail.com (N.U.); 2 Department of Chemistry, Biochemistry and Pharmaceutical Sciences, University of Bern, Freiestrasse 3, 3012 Bern, Switzerland; 3 KU Leuven, Department of Microbiology, Immunology and Transplantation, Laboratory of Virology and Chemotherapy, Rega Institute for Medical Research, Herestraat 49, 3000 Leuven, Belgium; leentje.persoons@rega.kuleuven.be (L.P.); dirk.daelemans@kuleuven.be (D.D.)

**Keywords:** diterpene, *ent*-kaurane, alkaloids, radical chemistry, ATRA, lactam, lactone, spiro compound, azide, pyrrolidine, cytotoxicity

## Abstract

A convenient strategy for molecular editing of available *ent*-kauranic natural scaffolds has been developed based on radical mediated C–C bond formation. Iodine atom transfer radical addition (ATRA) followed by rapid ionic elimination and radical azidoalkylation were investigated. Both reactions involve radical addition to the *exo*-methylenic double bond of the parent substrate. Easy transformations of the obtained adducts lead to extended diterpenes of broad structural diversity and artificial diterpene-alkaloid hybrids possessing lactam and pyrrolidine pharmacophores. The cytotoxicity of selected diterpenic derivatives was examined by in vitro testing on several tumor cell lines. The terpene-alkaloid hybrids containing *N*-heterocycles with unprecedented spiro-junction have shown relevant cytotoxicity and promising selectivity indexes. These results represent a solid basis for following research on the synthesis of such derivatives based on available natural product templates.

## 1. Introduction

**Figure 1:**
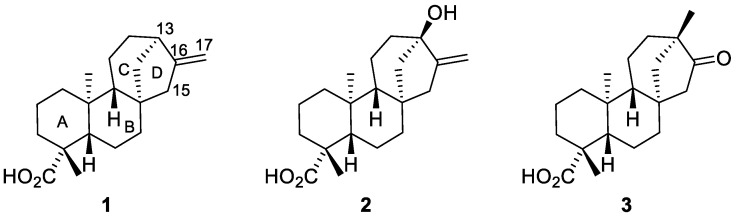
Structures of *ent*-kaurenoic acid **1**, steviol **2** and *iso*-steviol **3**.

The kauranic family of diterpenes is widespread in diverse plant sources and some representatives are readily available for large scale processing. The best known examples are *ent*-kaurenoic acid **1** and steviol **2** (Figure 1), which can be conveniently isolated from industrial crop derived products or residues. *Iso*-steviol **3** can also be included in this group, since it easily forms upon acidic hydrolysis of steviol **2** glycosides found in the *Stevia Rebaudiana* plant and is broadly used as an alternative non-caloric sweetener [1]. These compounds alone possess relevant biological activities [2,3,4], and numerous SAR studies have been reported based on their structural modifications [5,6,7]. In particular, chemical modification of steviol **2** and especially *iso*-steviol **3** has been well explored and delivered diverse compound libraries, basing on chemical reactivity of both carboxylic groups, as well as D-cycle functionalities [6,7].

*ent*-kaurenoic acid **1** has also been extensively involved in SAR studies and chemical modification was reported frequently at the level of the carboxylic functional group. The reactivity of the *exo*-methylenic Δ^16^-double bond was mostly addressed via the heteroatom addition processes or trivial oxidative transformations and only scarce data involve deeper skeletal modifications based on this functionality [8,9]. The *exo*-methylene moiety of acid **1** represents an additional opportunity for increasing molecular complexity based upon free radical processes. Free radical transformations are mild and versatile molecular editing tools, which have gained increasing popularity within the natural product chemistry community due to their tremendous potential for convenient late-stage functionalization of natural product scaffolds. Remote C–H functionalization is one example [10,11,12]. As a logical consequence, medicinal chemistry research can fully benefit from the structural diversity generated by free radical transformations of available natural product templates.

**Scheme 1:**
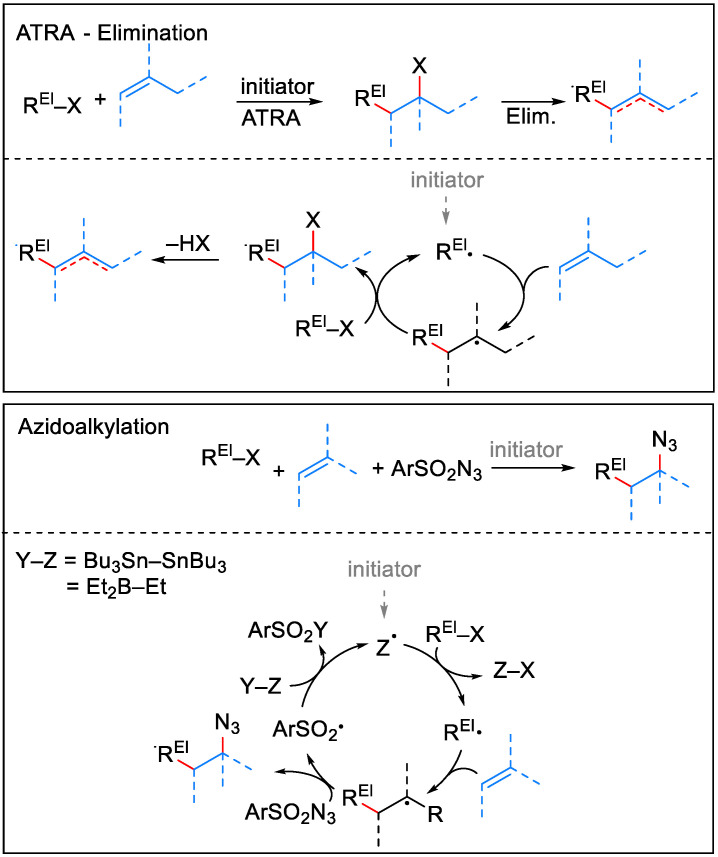
Mild transformation of alkenes via ATRA (-elimination) and azidoalkylation: useful tools for molecular editing (R^El^ = electrophilic C-residue).

In this context, we turned our attention to two mild free radical processes that proved efficient for the functionalization of olefinic bonds in other compound families: the atom transfer radical addition (ATRA) and the azidoalkylation reaction. ATRA are highly attractive transformations originating from the work of Kharasch, who discovered the addition of activated alkyl halides to alkenes [13,14,15]. This pioneering work was further refined into a valuable synthetic procedure [16] with a positive impact in diverse areas of chemistry [17]. Briefly, ATRA involves a starting alkyl halide with a weak C–X bond, which upon homolytic cleavage generates a carbon-centered radical prone to interaction with the olefinic moiety of the substrate in an addition process (Scheme 1, top). The resulting free radical abstracts the X-atom from the alkyl halide and the reaction chain perpetuates. Iodine-ATRA reactions leading to tertiary iodides are often followed by a rapid elimination of HI and conversion to alkenes [18,19]. The azidoalkylation reaction is closely related to the ATRA process but involves an extra azidating agent, usually a sulfonyl azide (Scheme 1, bottom) [20,21,22]. This reaction has been applied for the synthesis of several alkaloids [22,23,24] and its stereochemistry has been investigated in detail [25]. The formal result of both transformations is a carbo-functionalization of the alkene, including the simultaneous formation of both C–C and C–heteroatom bonds.

**Figure 2:**
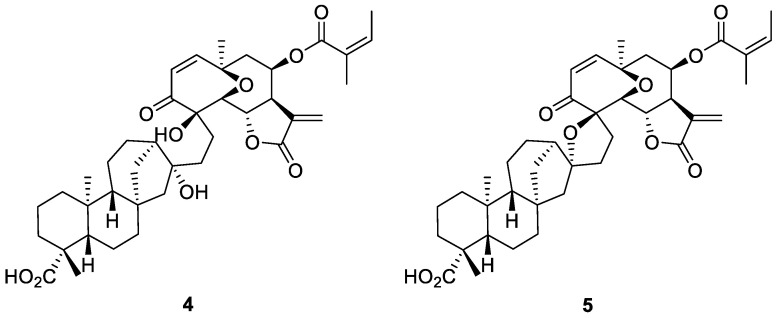
Structures of helikaurolides A (**4**) and B (**5**).

Such transformations are quite efficient and can be run under very mild reaction conditions, compatible with other functional groups present in the substrate. Therefore, it represents an ideal tool for molecular editing within SAR studies of complex molecules like *ent*-kauranes. It is worth mentioning that the *exo*-methylenic double bond in *ent*-kauranes is regarded as one of the crucial elements in the interaction with molecular targets [5] and the structural modifications of the diterpenic skeleton at the level of C (16)–C (17) carbon atoms can result in new properties of the corresponding derivatives. In this respect, C (17) alkylated *ent*-kauranes have been sporadically isolated from natural sources. A recent example relates to helikaurolides A (**4**) and B (**5**) (Figure 2)—unprecedented *ent*-kauranes, prenylated at C (17) with a densely functionalized sesquiterpenic unit [26]. Biogenetically, both **4** and **5** have been hypothesized based upon *ent*-kaurenic acid **1** alkylation with the sesquiterpenic unit. Therefore, radical C (17) alkylations represent an exact mimic of this biogenetical mechanism. Having this in mind, we report in the current paper the results of the synthesis and cytotoxic activity of new *ent*-kauranes based on radical additions to the *exo*-methylenic double bond of *ent*-kaurenoic acid **1** and its derivatives.

## 2. Results and Discussions

### 2.1. Radical Addition to ent-Kaurenoates

#### 2.1.1. Iodine-ATRA Reactions

**Scheme 2:**
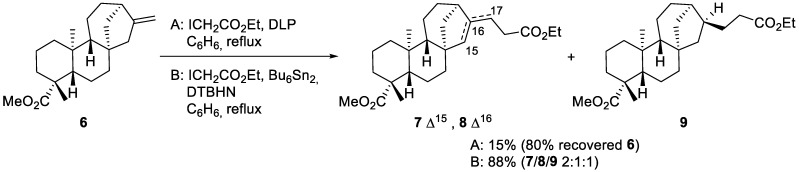
Iodine -ATRA of ethyl iodoacetate to methyl-*ent-kaur*enoate **6**.

Carboiodination is a process that formally adds an alkyl group and an iodine atom to the olefinic moieties. It is the simplest version of ATRA, and we started our work with carboiodination of methyl *ent*-kaurenoate **6**. It was prepared upon methylation of acid **1** with diazomethane. Ethyl iodoacetate was chosen as a readily available alkylating agent. The reaction was performed in refluxing benzene under inert atmosphere in the presence of dilauroyl peroxide (DLP) as an initiator (Scheme 2, Method A) [27].

DLP was gradually added to the reaction mixture in portions of 0.05 equivalents every 2 h during 24 h of reflux. Under these conditions, the conversion was sluggish and the starting ester **6** was recovered unchanged, along with minor amounts of esters **7**–**9** (15%). Their formation can be explained by the instability of the initially formed tertiary iodide, which eliminates HI to provide **7** and **8**. A hydrogen atom transfer, possibly involving DLP, can explain the formation of saturated **9**. The separation of these compounds was difficult and only the endocyclic isomer **7** was isolated in a sufficiently pure form to provide satisfactory spectral data.

In order to increase the conversion rate, we performed the reaction under the action of a hexabutylditin–di-*tert*-butyl hyponitrite (DTBHN) system, which showed good results in similar transformations [28]. The reaction resulted in the almost complete consumption of the starting material over 2 h at reflux and a mixture of esters **7**–**9** was produced with an 88% yield (Scheme 2, Method B, **7**/**8**/**9** 2:1:1). The formation of the tertiary iodide was not observed. Such eliminations of tertiary iodides has been described in the literature [18].

#### 2.1.2. Azidoalkylation

At this stage, it became clear that an efficient regiocontrol over HI elimination represents quite a challenging task for substrates with *exo*-methylenic double bonds. Therefore, we turned our attention to the azidoalkylation reaction, a transformation of special value from a SAR perspective due to the broad opportunities offered by the azide group for further transformation [29] such as 1,3-dipolar cycloaddition (click reaction) [30] and reduction to primary amines.

**Scheme 3:**
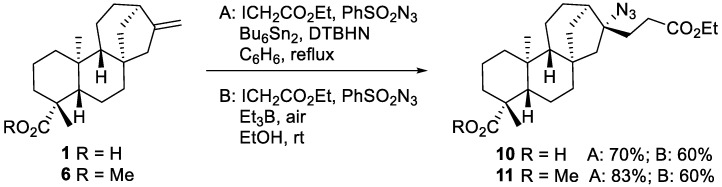
Azidoalkylation of *ent*-kaurenoic acid **1** and methyl-*ent*-kaurenoate **6**.

Azidoalkylation of acid **1** was performed under standard conditions using ethyl iodoacetate (radical precursor) and phenylsulfonyl azide (azidating agent) in the presence of hexabutylditin (chain transfer reagent) and DTBHN as an initiator [20]. The reaction proceeded well and the resulting azide **10** was isolated in 70% yield (Scheme 3, Method A). The free carboxyl group remained intact. Unfortunately, the purification of the product was difficult and total removal of residual tin contaminants was not possible. In order to avoid this problem, we investigated azidoalkylation of the corresponding methyl ester **6** under the same conditions. The azide **11** was isolated with an 83% yield without noticeable contamination by tin residues. To avoid the use of toxic hexabutylditin, the reaction was attempted using triethylborane as a mediator [31,32]. The compatibility of triethylborane with the free carboxylic functional group of **1** was expected to be an issue [33,34]. We decided to circumvent this problem by using a two-fold excess of Et_3_B and aqueous workup to hydrolyze acyloxyboron derivatives. The addition products **10** and **11** were both isolated in satisfactory 60% yields (Scheme 3, Method B).

**Scheme 4:**
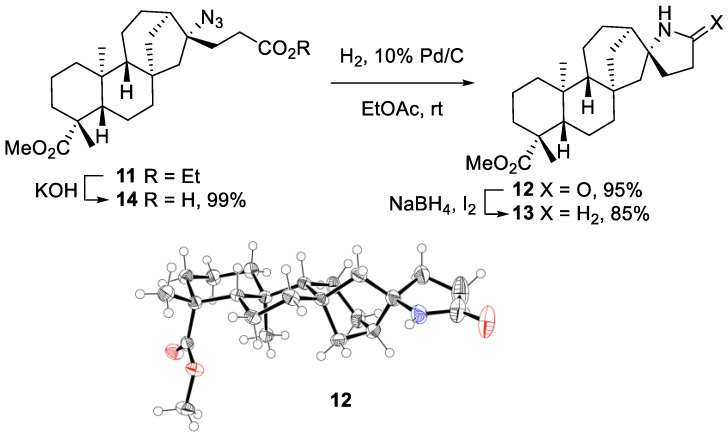
Conversion of the azidoalkylated product **11** into lactam **12**, pyrrolidine **13** and azido acid **14**. X-ray crystal structure of lactam **12** (ellipsoids drawn at 50% probability, CCDC 2023783).

To prepare the terpene-alkaloid hybrids, the product γ-azidoester **11** was converted to *N*-containg heterocycles. The hydrogenation of azide **11** went smoothly under 1 atmosphere of hydrogen pressure and Pd/C as a catalyst. The initially formed primary amine spontaneously formed the lactam **12** during the reduction (Scheme 4). Further reduction of the lactam **12** with NaBH_4_/I_2_ furnished the pyrrolidine **13** [35]. The spiro-cyclic system in both **12** and **13** represents a novel *ent*-kauranic-alkaloid hybrid that turned out to be a relevant pharmacophore, as revealed by cytotoxicity testing experiments described below. The relative stereochemistry of lactam **12** has been determined by single crystal X-ray crystallography (Scheme 4) [36]. These data confirmed indirectly the relative stereochemistry of starting azide **11**, showing that the addition of azide radicals occurred in a highly stereoselective way from the less hindered convex face of the tetracyclic *ent*-kauranic framework [25]. Furthermore, the ethyl ester moiety of azide **11** could be selectively hydrolyzed to provide the azido acid **14** in nearly quantitative yield.

**Scheme 5:**
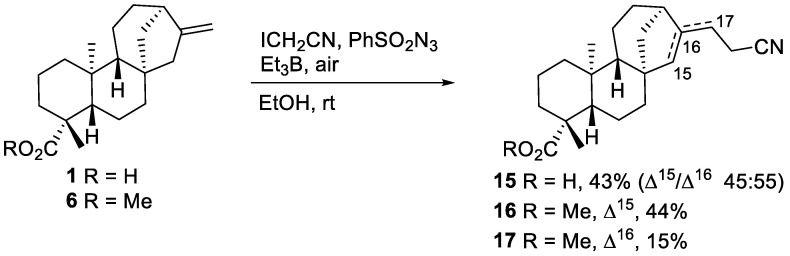
Azidoalkylation attempt of methyl-*ent*-kaurenoate **6** with iodoacetonitrile.

We also observed that the nature of the iodide represents an important factor affecting azidoalkylation of acid **1** and ester **6**. Our attempt to use iodoacetonitrile instead of ethyl iodoacetate did not result in any azidoalkylated product under Et_3_B initiation. The major reaction products are the unsaturated nitriles **15**–**17** (Scheme 5), similar in structure to esters **7** and **8**. This result is presumably due to the formation of an unstable iodide intermediate in the case of the nitrile, while the ester radical adduct reacts directly with the sulfonyl azide without the formation of a transient iodide. Indeed, iodoacetonitrile is a better iodine atom donor than the corresponding ester by about two orders of magnitude [37]. Trace amounts of an azide obtained from the azidoalkylation of acid **1** have been tentatively identified based on the characteristic ^13^C NMR peak at 72.25 ppm, corresponding to the C-N_3_ carbon atom. The isolation of this minor product turned out to be impossible using standard chromatography techniques.

### 2.2. Radical Addition to 15α-Hydroxy-ent-Kaurenoates

**Scheme 6:**
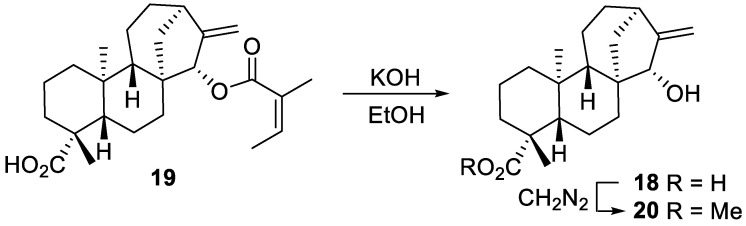
Preparation of methyl 15α-hydroxy-*ent*-kaurenoate **20**.

The modification of methyl 15α-hydroxy-*ent*-kaurenoate **20** was investigated next. The hydroxylated *ent*-kauranic acid derivative **18** comes together with *ent*-kaurenoic acid **1** in the sunflower vegetal mass as an angelic acid ester **19**. The latter can be easily isolated and hydrolyzed to provide pure **18** (Scheme 6). Methylation of **18** with diazomethane furnished quantitatively the methyl ester **20** used for the radical mediated modification experiments.

#### 2.2.1. Iodine ATRA Reactions

**Scheme 7:**
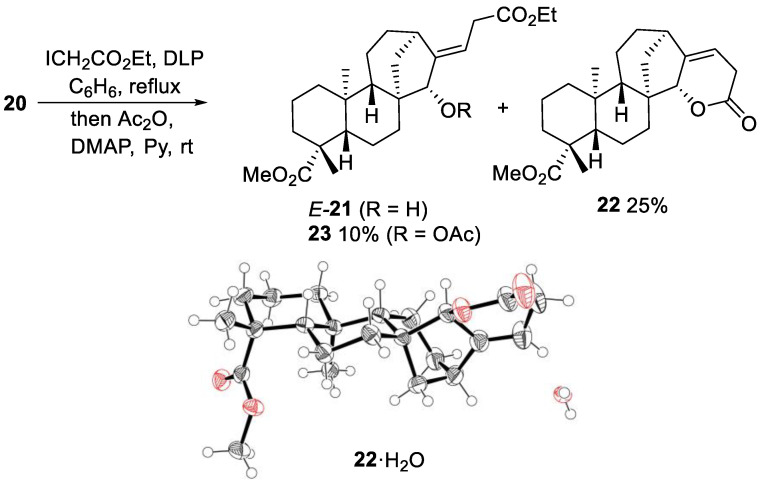
Addition of ethyl iodoacetate to **20**. X-ray crystal structure of lactone **22**·H_2_O (ellipsoids drawn at 50% probability, CCDC 2023785).

Upon heating the ester **20** with ethyl iodoacetate in benzene under DLP initiation, a mixture of two major unsaturated compounds *E*-**21** and **22** was obtained (Scheme 6). In line with similar ATRA experiments with substrates **1** and **6**, the hypothetically formed tertiary iodide is unstable, and rapid HI elimination leads to the unsaturated products [18]. Due to the presence of the alcohol moiety in **20**, lactonization of the *Z*-alkene resulting from the ATRA-elimination process occurred spontaneously under these reaction conditions leading to the unsaturated lactone **22**. The *E*-alkene cannot cyclize and the ester *E*-**21** is formed. Chromatographic separation of *E*-**21** from lactone **22** proved to be difficult due to their similar retention factors. Acetylation of the mixture of **21** and **22** with acetic anhydride resulted in the formation of acetate **23** which could be easily separated by chromatography and provided satisfactory analytical data for full characterization (Scheme 7). The structure of lactone **22** was obtained by single crystal X-ray crystallography (Scheme 7) [36]. The basic challenge at this stage was the demonstration of the *E*-configuration of the exocyclic double bond in acetate **23** and indirectly in alcohol **21**. The convincing proof came from the experiments on azidoalkylation of the allylic alcohol **20** (see below).

#### 2.2.2. Azidoalkylation

**Scheme 8:**
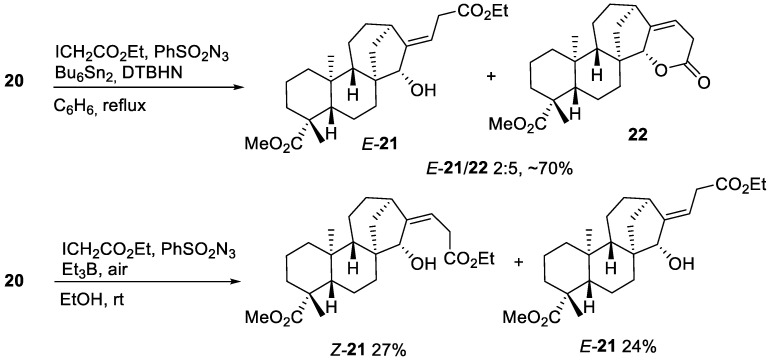
Iodine-ATRA between methyl 15α-hydroxy-*ent*-kaurenoate **20** and ethyl iodoacetate.

The azidoalkylation process was investigated under similar conditions that brought about optimal conversion of methyl *ent*-kaurenoate **6** to the azide **11**. Upon treatment of **20** with ethyl iodoacetate in the presence of hexabutylditin–DTBHN and phenylsulfonyl azide, an optimal 90% conversion of starting material was achieved after 5 h of heating at reflux in benzene (Scheme 8). However, no azide was detected but the already known ester *E*-**21** and lactone **22** were obtained. When the same reaction was run at room temperature under triethylborane initiation, again no azide was formed, and a mixture of unsaturated esters *E*-**21** and *Z*-**21** was obtained (Scheme 8). Interestingly, the lower reaction temperature impedes the lactonization of *Z*-**21** and its isolation was possible. The absence of the azidoalkylated product is presumably a consequence of the increased steric hindrance and decreased nucleophilicity of the hydroxylated radical adduct relative to the non-hydroxylated one, leading to **11**.

**Figure 3:**
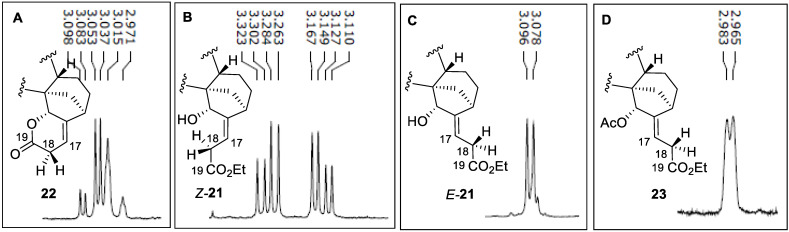
^1^H NMR spectra sections of lactone **21** (**A**), ester **24** (**B**), ester **22** (**C**), and acetate **23** (**D**).

Having accessed all four compounds *E*-**21**, *Z*-**21**, **22**, and **23** of sufficient purity, we were able to draw conclusions on their stereochemistry based on the ^1^H NMR spectra, in particular upon examining the splitting character of C (18) methylenic protons adjacent to the carboxylic group in these compounds (Figure 3).

First, these methylenic protons in **22** resonate at 3.02 ppm (Figure 3A) and show a relevant geminal coupling, which is due to the rigid cyclic structure and strong influence of the adjacent oxygenated moieties. The vicinal coupling with the vinylic proton is in accordance with dihedral angles observed in X-ray pictures. The ^1^H NMR spectrum of *Z*-**21** shows a similar splitting profile of the methylenic group at C (18) (Figure 3B, 3.11–3.32 ppm) to that observed in lactone **22**, demonstrating the proximity of this group to the hydroxyl at C (15) position, leading to a larger chemical shift difference between two geminal protons at C (18). On the other hand, both C (18) methylenic protons in ester *E*-**21** and acetate **23** are almost equivalent due to the conformational mobility of the lateral chain and impeded interaction with the oxygenated functional group. Their NMR peaks represent doublets, resonating at 3.08 ppm (Figure 3C) and 2.97 ppm (Figure 3D), respectively, and having an identical coupling constant of 7.3 Hz with vinylic protons from C (17).

**Scheme 9:**
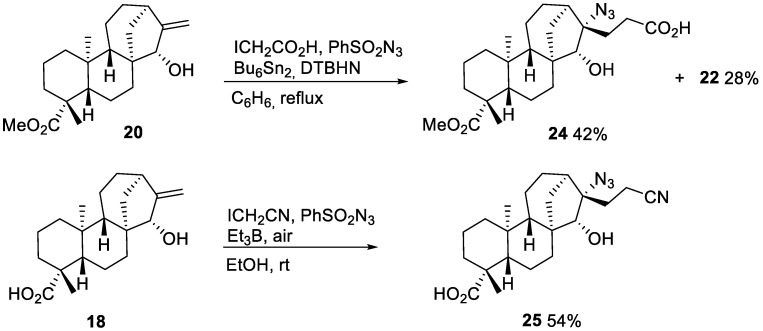
Azidoalkylation of methyl 15-α-hydroxy-*ent*-kaurenoate **20** and 15-α-hydroxy-*ent*-kaurenoic acid **18** with iodoacetic acid and iodoacetonitrile.

Interestingly, an attempt to run the azidoalkylation process with iodoacetic acid instead of its ethyl ester led to the azide **24** and lactone **22** (Scheme 9). The azidoalkylation was also achieved with iodoacetonitrile as the radical precursor. Performing the reaction with triethylborane as radical initiator was compatible with the free carboxyl group in the starting *ent*-kaurane **18** and resulted in product **25**, opening up more opportunities for further functionalization.

**Scheme 10:**
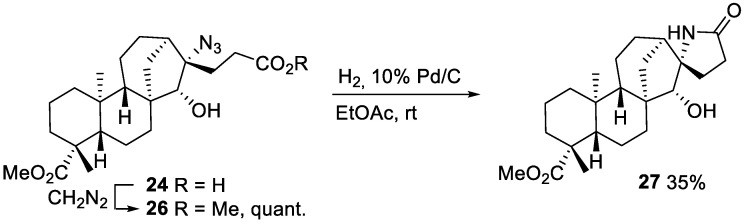
Conversion of the azidoalkylated acid **24** into ester **26** and lactam **27**.

Further transformations of azido-acid **24** included its methylation with diazomethane to ester **26**, which could be converted into lactam **27** during reduction of the azide under hydrogenation conditions (Scheme 10).

### 2.3. Cytotoxicity Testing

The described processes resulted in a small library of new compounds with modified *ent*-kaurane skeletons, and their cytotoxic activity has been investigated. Proliferation assays have been performed in seven different tumor cell lines as shown in Table 1: Capan-1 (pancreatic adenocarcinoma), HCT-116 (colorectal carcinoma), NCI-H460 (lung carcinoma), DND-41 (acute lymphoblastic leukemia), HL-60 (acute myeloid leukemia), K-562 (chronic myeloid leukemia), and Z-138 (non-Hodgkin lymphoma). A parallel cytotoxicity assay was performed on non-cancerous retina cells (hTERT RPE-1) in order to estimate the selectivity of the investigated compounds towards tumor cells. The inhibition of cell proliferation was calculated as IC_50_ by interpolation based on the semi-log dose response. Docetaxel and staurosporine have been included as positive controls. The data in Table 1 show that a significant portion of the investigated compounds demonstrate relevant antitumoral activity. In particular, compounds **11**–**13**, **22**, **26**, and **27** were active with IC_50_ values below 1 μM concentrations. We were also pleased to observe a selective activity for some compounds, based on much higher IC_50_ for normal non-cancerous cells. The calculated selectivity indices (SI), i.e., the ratio of the IC_50_ for normal cells to the IC_50_ for the corresponding tumor cells [38], are higher than the threshold value of 3 for several samples (see table in the supplementary material for details). The highest selectivity indices were obtained for compounds **11**, **12**, **13**, **26**, and **27**, with IC_50_ values for retina (hTERT RPE-1) cells up to 43-fold higher than those for cancerous cells. These data are indicative of a potential therapeutic index for these compounds, which can be explored in subsequent in vivo toxicity tests.

**Figure 4:**
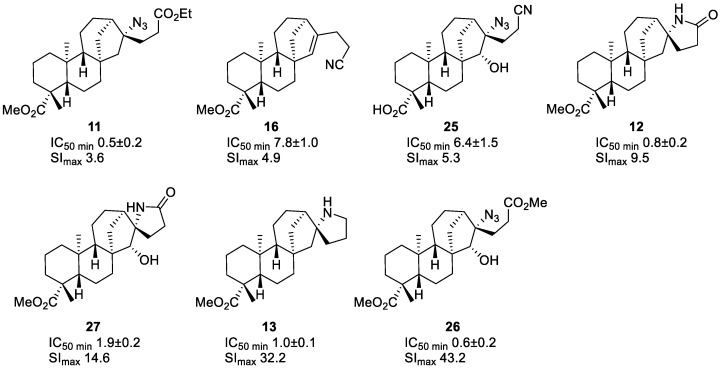
Relevant cytotoxic activity parameters for selected compounds.

From a structural topology point of view, the most active compounds show some relevant similarities (Figure 4). First of all, most of the active compounds include nitrogen containing functional groups. The second structural convergence relates to the cyclic backbone, which includes an extra cycle to the natural *ent*-kauranic framework. Exceptions are azides **11** and **26**. Finally, three of the four most relevant cytotoxic compounds with high selectivity towards tumor cells are heterocycles **12**, **13**, and **27** with a spirocyclic fragment. To the best of our knowledge, such derivatives of *ent*-kauranic compounds have never been reported in SAR studies. The activity levels of these compounds is much higher than that of the reference drug Cisplatin (IC_50_ 16.5 for HCT-116 and 5.33 for NCI-H460) and in the same range as the currently marketed drug midostaurine, with IC_50_ 0.237 for HCT-116 and 0.43 for NCI-H460 [39]. In fact, compounds **12** and **27** share the same *γ*-lactam unit with both natural staurosporine and synthetic midostaurine. Although this pharmacophore is becoming increasingly more studied [40], little is known about the properties of γ-spiro-1-lactams, which are extremely rare in the literature. The same relates to their mechanism of interaction with cellular targets [41]. Following in vivo toxicity studies can reveal the therapeutic potential of the reported new *ent*-kauranic derivatives.

## 3. Materials and Methods

### 3.1. Chemistry General Section

IR spectra were recorded on a Perkin–Elmer (Beaconsfield, UK) Spectrum 100 FT-IR spectrophotometer using the universal ATR sampling accessory. NMR spectra were recorded in CDCl_3_ on a Bruker (Rheinstetten, Germany) Avance III spectrometer (400.13 and 100.61 MHz). Chemical shifts (*δ*) are reported in parts per million (ppm) downfield from tetramethylsilane Si (CH_3_)_4_ (*δ* = 0.00 for ^1^H NMR spectra) using residual solvent signal as an internal standard: *δ* singlet 7.26 (^1^H), triplet 77.0 (^13^C) for CDCl_3_ or *δ* singlet 4.87 (^1^H), septet 49.0 (^13^C) for CD_3_OD. Multiplicities are given as s (singlet), d (doublet), t (triplet), q (quadruplet), m (multiplet), and bs (broad signal). Coupling constant (*J*) is reported in Hz. In case of mix of products, selected values for ^1^H NMR were reported (as specified in the description). In ^13^C NMR spectra, the peak positions are reported to one decimal unless the difference in chemical shift between two signals (for example for regio-/diastereoisomers) is small and required two decimals. Optical rotations were measured in CHCl_3_ or MeOH on a Jasco DIP 370 polarimeter, using a 5-cm cell. GC-MS experiments were performed on an Agilent 7890A (Wilmington, DE, USA) chromatograph equipped with a quadrupole MS detector MSD 5975C and an HP-5ms capillary column (30 m/0.25 mm/0.25 μm). Analytical TLC was performed on Fluka Silica Alu foils. Usual workup included dilution with brine and extraction with Et_2_O. The organic phases have been dried over Na_2_SO_4_, filtered and the solvent evaporated under reduced pressure to provide the crude reaction product. Column chromatography (CC) was performed with Fluka Kieselgel 60 silica gel. All solvents were of reagent grade and other reagents have been used as received from suppliers. DLP was added in aliquot portions every 2 h to the solution of the corresponding olefin and iodide. Addition of Et_3_B solutions (commercial) to the reaction mixtures was performed via a syringe with the needle immersed into the reaction mixture to avoid direct contact of Et_3_B drops with air. ATRA reactions have been performed with ethyl iodoacetate unless otherwise specified. Benzene and THF have been dried according to standard procedures and distilled before use.

### 3.2. Crystal-Structure Determination

The crystals were mounted in air at ambient conditions. All measurements were made on an *Oxford Diffraction SuperNova* area-detector diffractometer [42] using mirror optics monochromated Mo *K*α radiation (λ = 0.71073 Å) and Al filtered [43]. Data reduction was performed using the *CrysAlisPro* [42] program. The intensities were corrected for Lorentz and polarization effects, and an absorption correction based on the multi-scan method using SCALE3 ABSPACK in *CrysAlisPro* [42] was applied. Data collection, refinement parameters and other specific experimental details are given in the Supplementary Materials. The structure was solved by direct methods using *SHELXT* [44], which revealed the positions of all non-hydrogen atoms of the title compound. The non-hydrogen atoms were refined anisotropically. All H-atoms were placed in geometrically calculated positions and refined using a riding model where each H-atom was assigned a fixed isotropic displacement parameter with a value equal to 1.2Ueq of its parent atom. Refinement of the structure was carried out on *F*^2^ using full-matrix least-squares procedures, which minimized the function Σw (F_o_^2^–F_c_^2^)^2^. The weighting scheme was based on counting statistics and included a factor to downweight the intense reflections. All calculations were performed using the *SHELXL-2014/7* [45] program in OLEX2 [46].

### 3.3. Cytotoxicity Testing

#### 3.3.1. Cell Culture and Reference Compounds

All cell lines (HL-60, K-562, Z-138, Capan-1, HCT-116, NCI-H460 and hTERT RPE-1) were acquired from the American Type Culture Collection (ATCC, Manassas, VA, USA), except for the DND-41 cell line, which was purchased from the Deutsche Sammlung von Mikroorganismen und Zellkulturen (DSMZ Leibniz-Institut, Braunschweig, Germany). All cell lines were cultured as recommended by the suppliers. Culture media were purchased from Gibco Life Technologies, USA, and supplemented with 10% fetal bovine serum (HyClone, GE Healthcare Life Sciences, Logan, UT, USA). Reference inhibitor compounds staurosporine and docetaxel were obtained from Selleckchem (Munich, Germany). All stock solutions were prepared in DMSO.

#### 3.3.2. Cell Proliferation Assays

Adherent cell lines Capan-1, HCT-116 and NCI-H460 cells were seeded at a density between 500 and 1500 cells per well in 384-well clear-bottomed tissue culture plates (Greiner). After overnight incubation, cells were treated with the test compounds at seven different concentrations ranging from 100 to 6.4 × 10^−3^ µM. Suspension cell lines DND-41, HL-60, K-562, and Z-138 were seeded at densities ranging from 2500 to 5500 cells per well in 384-well clear-bottomed tissue culture plates containing the test compounds at the same seven concentration points. The plates were incubated at 37 °C and monitored for 72 h in an IncuCyte^®^ device (Essen BioScience Inc., Ann Arbor, MI, USA) for real-time imaging. Images were taken every 3 h, with one field imaged per well under 10× magnification. Cell growth was then quantified based on the percent cellular confluence as analysed by the IncuCyte^®^ image analysis software and used to calculate IC_50_ values by linear interpolation. All tests have been performed in duplicate and the average values have been calculated. The corresponding mean values and deviations are provided in Table 1.

### 3.4. Experimental Section

DLP-mediated ATRA to methyl *ent*-kaurenoate

Methyl *ent*-kaurenoate **6** (50 mg, 0.16 mmol) in dry C_6_H_6_ (5 mL) was treated with ethyl iodoacetate (28 µL, 0.24 mmol) and DLP (3 mg, 0.008 mmol). The reaction mixture was heated on reflux under N_2_ for 24 h. The mixture was purified by CC (hexane/EtOAc 98:2) and gave unchanged starting material (40 mg, 80%) and a mixture of compounds **7**, **8** and **9** (10 mg, 15%).

Ester **7**

Pale yellow viscous oil. ${\left[\alpha \right]}_{D}^{20}$ = −45.8 (c = 8.13, CHCl_3_). IR (ν, cm^−1^): 3804, 3736, 3674, 3608, 2927, 1728, 1445, 1231, 1156. ^1^H NMR (400 MHz, CDCl_3_) δ_H_: 5.07 (s, 1H, CH-15), 4.13 (q, *J* = 7.1 Hz, 2H, -CO_2_CH_2_CH_3_), 3.63 (s, 3H, -CO_2_CH_3_), 2.44–2.49 (m, 2H, -CH_2_-COOEt), 1.25 (t, *J* = 7.1 Hz, 3H, -CO_2_CH_2_CH_3_), 1.16 (s, 3H, CH_3_-20), 0.83 (s, 3H, CH_3_-22), 2.38–0.93 (m, 21H). ^13^C NMR (100 MHz, CDCl_3_) δ_C_: 178.0, 173.4, 145.1, 134.4, 60.2, 56.9, 51.0, 49.0, 47.8, 43.9, 43.8, 40.8, 39.6, 39.5, 38.2, 32.4, 29.7, 28.7, 25.4, 25.3, 20.8, 19.2, 18.9, 15.2, 14.2. GCMS *m*/*z* calculated for [C_25_H_38_O_4_]^+^: 402.28; found: 402.3.

Ester **8**

Mixture of 2 diastereomers. IR (v, cm^−1^): 3674, 2931, 1728, 1449, 1234, 1171, 1150. ^1^H NMR (400 MHz, CDCl_3_) selected values, δ_H_: 4.15–4.09 (m, 2H, -CO_2_CH_2_CH_3_), 3.64 (s, 3H, -CO_2_CH_3_), 1.25 (m, 3H, -CO_2_CH_2_CH_3_), 1.17 (m, 3H, CH_3_-20), 0.82 (m, 3H, CH_3_-22). ^13^C NMR (100 MHz, CDCl_3_) selected values, δ_C_: 178.04/178.03, 172.57/172.41, 149.98/148.94, 110.39/109.94, 60.36, 57.12/57.07, 55.17/55.10, 51.03, 49.32/46.21, 43.84, 43.59/43.48, 41.41/41.19, 40.80, 39.63/39.47, 39.41/39.27, 38.12, 34.90/34.68, 33.06/30.83, 28.73, 21.96, 19.16, 18.76/18.45, 15.45/15.41, 14.21/14.18. GCMS *m*/*z* calculated for C_25_H_38_O_4_: 402.28; found: 402.3.

Ester **9**

Colorless oil. IR (ν, cm^−1^): 3750, 2921, 1729, 1506, 1448, 1370, 1191, 1042. ^1^H NMR (400 MHz, CDCl_3_) δ_H_: 4.12 (q, *J* = 7.9 Hz, 2H, -CO_2_CH_2_CH_3_), 3.63 (s, 3H, -CO_2_CH_3_), 2.86–2.60 (m, 1H), 2.29 (m, 2H, CH_2_-17), 1.25 (t, *J* = 7.1 Hz, 3H, -CO_2_CH_2_CH_3_), 1.16 (s, 3H, CH_3_-20), 0.81 (s, 3H, CH_3_-22), 2.18–0.79 (m, 23H). ^13^C NMR (100 MHz, CDCl_3_) δ_C_: 178.10, 174.03, 60.17, 57.15, 56.61, 51.05, 46.74, 44.41, 43.89, 42.22, 40.87, 40.51, 40.13, 39.48, 38.49, 38.21, 34.09, 28.77, 26.66, 25.88, 22.24, 19.19, 19.14, 15.42, 14.28. GCMS *m*/*z* calculated for C_25_H_40_O_4_: 404.29; found: 404.3.

Bu_6_Sn_2_-mediated ATRA to methyl *ent*-kaurenoate

Methyl *ent*-kaurenoate **6** (50 mg, 0.158 mmol) in dry C_6_H_6_ (3 mL) was treated under N_2_ with ethyl iodoacetate (37.4 µL, 0.316 mmol), Bu_6_Sn_2_ (120 µL, 0.237 mmol), and DTBHN (0.8 mg, 0.005 mmol). The reaction mixture was refluxed for 2 h. The crude product was purified by CC (hexane/EtOAc 98:2) and gave a 2:1:1 mixture of compounds **7**, **8**, and **9** (61 mg, 88% total yield). Separation of this mixture on a silver nitrate impregnated silica afforded a pure sample of ester **7** (45% yield), along with esters **8** and **9** (22% and 21% yield, respectively).

Bu_6_Sn_2_-mediated ATRA to *ent*-kaurenoic acid

*ent*-Kaurenoic acid **1** (87 mg, 0.29 mmol) in dry benzene (2 mL) was treated under N_2_ with ethyl iodoacetate (51.5 µL, 0.44 mmol), PhSO_2_N_3_ (159 mg, 0.87 mmol), Bu_6_Sn_2_ (222 µL, 0.44 mmol), and DTBHN (1.5 mg, 0.087 mmol). The reaction mixture was refluxed for 2 h and after this time the crude product was purified by CC (pentane/EtOAc 90:10) giving compound **10**, slightly contaminated with tin compounds (NMR). This impurified product was methylated with an ethereal solution of diazomethane and purified by CC (hexane/EtOAc 98:2) to afford azide **11** (90 mg, 70%) as a colorless oil.

Azide **11**

Colorless viscous oil. ${\left[\alpha \right]}_{D}^{20}$ = −79.5 (c = 0.84, CHCl_3_). IR (ν, cm^−1^): 2944, 2873, 2096, 1727, 1502. ^1^H NMR (400 MHz, CDCl_3_) δ_H_: 4.12 (q, *J* = 7.2 Hz, 2H, -CO_2_CH_2_CH_3_), 3.62 (s, 3H, -CO_2_CH_3_), 2.42 (t, *J* = 7.9 Hz, 2H, -CH_2_-COOEt), 1.25 (t, *J* = 7.2 Hz, 3H, -CO_2_CH_2_CH_3_), 1.14 (s, 3H, CH_3_-20), 0.80 (s, 3H, CH_3_-22), 2.06–0.75 (m, 23H). ^13^C NMR (100 MHz, CDCl_3_) δ_C_: 177.9, 173.4, 73.1, 60.5, 56.7, 55.8, 51.5, 51.1, 44.7, 44.3, 43.7, 41.4, 40.6, 39.4, 38.0, 37.7, 30.3, 29.7, 28.7, 26.1, 22.0, 19.0, 18.5, 15.3, 14.2. GCMS *m*/*z* calculated for [C_25_H_39_N_3_O_4_]: 445.28; found: 402.3 (M-HN_3_).

Bu_6_Sn_2_-mediated ATRA to methyl *ent*-kaurenoate

Methyl *ent*-kaurenoate **6** (100 mg, 0.36 mmol) in dry benzene (2 mL) was treated under N_2_ with ethyl iodoacetate (64 µL, 0.54 mmol), PhSO_2_N_3_ (200 mg, 1.08 mmol), Bu_6_Sn_2_ (272 µL, 0.54 mmol), and DTBHN (2 mg, 0.01 mmol). The reaction mixture was refluxed for 2 h. The crude product was purified by column chromatography (hexane/EtOAc 98:2) and gave the azide **11** (133 mg, 83%).

Et_3_B-mediated ATRA to *ent*-kaurenoic acid

*ent*-Kaurenoic acid **1** (44 mg, 0.146 mmol) in dry EtOH (2 mL) was treated under N_2_ with ethyl iodoacetate (35 µL, 0.292 mmol), PhSO_2_N_3_ (80 mg, 0.438 mmol), Et_3_B (0.6 mL, 0.584 mmol). The reaction was stirred at room temperature overnight then diluted with brine and worked up. The crude product was purified by CC (hexane/EtOAc 94:6) giving pure compound **10** (39 mg, 60%).

Azide **10**

Pale yellow viscous oil. ${\left[\alpha \right]}_{D}^{20}$ = −69.2 (c = 1.9, CHCl_3_). IR (ν, cm^−1^): 2935, 2900, 2872, 2848, 2626, 2098, 1734, 1693. ^1^H NMR (400 MHz, CDCl_3_) δ (ppm) 4.14 (q, *J* = 7.1 Hz, 2H, -CO_2_CH_2_CH_3_), 2.43 (t, *J* = 8.1 Hz, 2H, -CH_2_-COOEt), 1.26 (t, *J* = 7.1 Hz, 3H, -CO_2_CH_2_CH_3_), 1.22 (s, 3H, CH_3_-20), 0.93 (s, 3H, CH_3_-22), 2.15–0.73 (m, 23H). ^13^C NMR (100 MHz, CDCl_3_) δ (ppm) 184.9, 173.4, 73.1, 60.6, 56.7, 55.8, 51.5, 44.7, 44.3, 43.7, 41.4, 40.5, 39.6, 37.7, 37.6, 30.3, 29.7, 28.9, 26.1, 21.8, 18.9, 18.5, 15.4, 14.2.

Et_3_B-mediated ATRA to methyl *ent*-kaurenoate

Methyl *ent*-kaurenoate **6** (50 mg, 0.16 mmol) in dry EtOH (2 mL) was treated under N_2_ with ethyl iodoacetate (39 µL, 0.33 mmol), PhSO_2_N_3_ (87 mg, 0.47 mmol) and Et_3_B (0.47 mL). The reaction mixture was stirred for 2 h at room temperature. The crude product was purified by column chromatography (pentane/EtOAc 98:2) and gave the azide **11** (43 mg, 60%).

Lactam **12**

A solution of azide **11** (53 mg, 0.12 mmol) and 10% Pd/C (10% *w*/*w*) in dry EtOAc (2 mL) was stirred for 48 h at room temperature under H_2_ (1 atm). The catalyst was filtered off, the solvent was removed under reduced pressure, and the crude purified by column chromatography (CH_2_Cl_2_/MeOH 90:10) giving lactam **12** (42 mg, 95%) as a white powder.

White powder. M.p. 150–152 °C, from CH_2_Cl_2_. ${\left[\alpha \right]}_{D}^{20}$ = −77.4 (c = 4.50, CHCl_3_). IR (ν, cm^−1^): 3676, 3209, 2937, 2298, 1712, 1683. ^1^H NMR (400 MHz, CDCl_3_) δ_H_: 6.68 (s, 1H, -NH), 3.63 (s, 3H, -CO_2_CH_3_), 2.30 (m, 2H, CH_2_-17), 2.05 (d, *J* = 11.9 Hz, 2H, CH_2_-18), 1.15 (s, 3H, CH_3_-20), 0.81 (s, 3H, CH_3_-22), 2.36–0.79 (m, 21H). ^13^C NMR (100 MHz, CDCl_3_) δ_C_: 177.9, 176.8, 67.2, 57.6, 56.9, 55.6, 51.1, 46.6, 44.9, 43.8, 41.8, 40.7, 39.4, 38.9, 38.1, 31.1, 30.0, 28.7, 27.1, 22.0, 19.1, 18.8, 15.4.

Pyrrolidine **13**

To a solution of lactam **12** (50 mg, 0.13 mmol) in THF (4 mL) were added NaBH_4_ (20 mg, 0.53 mmol) and I_2_ (102 mg, 0.4 mmol). The reaction mixture was refluxed for 8 h, then cooled at 0 °C and excess hydride quenched with 3N HCl solution, then neutralized and worked up as usual. The crude product was purified by CC (CH_2_Cl_2_/MeOH 90:10) giving compound **13** (40 mg, 85%) as a colorless oil.

Colorless oil. ${\left[\alpha \right]}_{D}^{20}$ = −73.6 (c = 2.74, CHCl_3_). IR (ν, cm^−1^): 3424, 2940, 2871, 2744, 2483, 1722, 1599. ^1^H NMR (400 MHz, CDCl_3_) δ (ppm) 8.46 (s, 1H, NH) 3.63 (s, 3H, -CO_2_CH_3_), 3.43 (bs, 2H, CH_2_-19), 2.65 (s, 1H, CH-13), 1.15 (s, 3H, CH_3_-20), 0.81 (s, 3H, CH_3_-22), 2.09–0.78 (m, 24H). ^13^C NMR (100 MHz, CDCl_3_) δ (ppm) 177.9, 76.8, 56.7, 55.7, 52.4, 51.1, 45.7, 43.8, 43.3, 42.7, 40.7, 40.3, 39.5, 38.5, 38.1, 33.0, 28.6, 26.9, 22.7, 22.1, 19.0, 18.6, 15.3.

Azide **14**

Azide **11** (50 mg, 0.11 mmol) was dissolved in EtOH and treated with KOH (31.4 mg, 0.56 mmol) on reflux for 2 h. Then, the reaction mixture was diluted with brine and extracted with Et_2_O and ethereal extract washed with 10% sol. H_2_SO_4_, sat. sol. NaHCO_3_, brine and dried over Na_2_SO_4_. The crude product was purified by CC (petroleum ether/EtOAc 90:10), providing the azide **14** as colorless oil (45 mg, 98%).

Colorless oil. ${\left[\alpha \right]}_{D}^{20}$ = −85.6 (c = 4.50, CHCl_3_). IR (ν, cm^−1^): 3855, 3668, 2942, 2098, 1724, 1715, 1446, 1236, 1154, 1066, 1051, 774. ^1^H NMR (400 MHz, CDCl_3_) δ_H_: 3.64 (s, 3H, -CO_2_CH_3_), 2.51 (t, *J* = 7.97 Hz, 2H, CH_2_-17,), 1.16 (s, 3H, CH_3_-20), 0.82 (s, 3H, CH_3_-22), 2.16–0.99 (m, 23H). ^13^C NMR (100 MHz, CDCl_3_) δ_C_: 178.6, 178.0, 72.9, 56.7, 55.8, 51.5, 51.2, 44.8, 44.3, 43.7, 41.4, 40.6, 39.4, 38.0, 37.7, 29.9, 29.5, 28.7, 26.2, 22.0, 19.0, 18.5, 15.3.

Et_3_B-mediated ATRA of iodoacetonitrile to *ent*-kaurenoic acid.

*ent*-Kaurenoic acid **1** (190.0 mg, 0.63 mmol) in 3.5 mL EtOH was treated under N_2_ with iodoacetonitrile (91 μL, 1.25 mmol), PhSO_2_N_3_ (345.0 mg, 1.88 mmol) and Et_3_B (1.9 mL, 1.88 mmol) at room temperature overnight. The crude product was purified by FC (petroleum ether/EtOAc 90:10) and provided the isomeric mixture **15** (95 mg, 42%, ∆^15^: ∆^16^ = 45:55, NMR data).

Mixture of nitriles **15**

IR (ν, cm^−1^): 3671, 2923, 2854, 2666, 1696, 1469, 1445, 1413. ^1^H NMR (400 MHz, CDCl_3_) selected values, δ_H_: 5.23 (s, CH-15), 5.18–5.14 (m, CH-17), 2.98 (s, 2xCH_2_-18), 2.15 (m, CH_2_-17 ∆^15^), 1.24, 1.23 (s, CH_3_-20), 0.93, 0.95 (s, CH_3_-22); ^13^C NMR (100 MHz, CDCl_3_) selected values, δ_C_ (major ∆^16^): 184.04, 153.35, 118.38, 105.82, 56.99, 54.94, 45.94, 44.02, 41.20, 40.67, 39.67, 39.30, 37.75, 32.87, 28.92, 21.79, 19.05, 18.41, 17.04, 15.59; (minor ∆^15^): 184.26, 142.85, 136.04, 119.73, 56.72, 49.22, 47.42, 43.76, 43.40, 40.66, 39.85, 39.26, 37.79, 28.94, 25.96, 25.32, 20.67, 19.05, 18.95, 15.56, 15.38.

Et_3_B-mediated ATRA of iodoacetonitrile to methyl *ent*-kaurenoate

Methyl *ent*-kaurenoate **6** (180.0 mg, 0.56 mmol) in 3.5 mL EtOH was treated under N_2_ with iodoacetonitrile (82 μL, 1.13 mmol), PySO_2_N_3_ (313.0 mg, 1.7 mmol), and Et_3_B (1.7 mL, 1.7 mmol) at room temperature overnight. The crude product was purified by CC (petroleum ether/EtOAc 95:5) and provided the mixture of unsaturated derivatives **16** and **17** (117 mg, 58%, **16**/**17** = 3:1, NMR data). This mixture was submitted to a repetitive separation on a silver nitrate impregnated silica column and individual nitriles slightly contaminated with a second isomer were obtained as a colorless oils.

Nitrile **16**

Colorless oil. ${\left[\alpha \right]}_{D}^{20}$ = −48.8 (c = 4.6, CHCl_3_). IR (ν, cm^−1^): 2932, 2848, 1723, 1639, 1468, 1446, 1232, 1158. ^1^H NMR (400 MHz, CDCl_3_) δ_H_: 5.22 (s, 1H, CH-15), 3.63 (s, 3H, -CO_2_CH_3_), 2.49–2.38 (m, 2H, CH_2_-18), 1.15 (s, 3H, CH_3_-20), 0.99 (s, 3H, CH_3_-22), 2.19–0.78 (m, 21H). ^13^C NMR (100 MHz, CDCl_3_) δ_C_: 178.0, 142.8, 136.0, 119.8, 56.7, 51.1, 49.2, 47.4, 43.8, 43.8, 43.4, 40.7, 39.6, 39.3, 38.1, 28.7, 25.9, 25.3, 20.7, 19.1, 18.9, 15.5, 15.2.

Nitrile **17**

Colorless oil. ${\left[\alpha \right]}_{D}^{20}$ = −100.8 (c = 3.4, CHCl_3_). IR (ν, cm^−1^): 2931, 2855, 1723, 1461, 1447, 1150. ^1^H NMR (400 MHz, CDCl_3_) δ_H_: 5.17–5.13 (m, 1H, CH-17), 3.64 (s, 3H, -CO_2_CH_3_), 3.04 (m, 2H, CH_2_-18), 2.84 (bs, 1H), 2.21–1.96 (m, 1H), 1.17 (s, 3H, CH_3_-20), 0.83 (s, 3H, CH_3_-22), 2.06–0.79 (m, 19H). ^13^C NMR (100 MHz, CDCl_3_) δ_C_: 178.1, 152.5, 118.9, 106.5, 57.2, 55.1, 51.3, 49.3, 44.0, 43.8, 41.2, 40.9, 39.7, 39.6, 38.2, 30.8, 29.7, 28.9, 22.1, 19.3, 18.9, 17.2, 15.7.

DLP-mediated ATRA to methyl 15α-hydroxy-*ent*-kaurenoate

Methyl 15α-hydroxy-*ent*-kaurenoate **20** (56 mg, 0.17 mmol) in 3.7 mL benzene was treated under N_2_ with ethyl iodoacetate (60 µL, 0.507 mmol) and DLP (3.4 mg, 0.009 mmol). The reaction was refluxed for 24 h and after this time the crude product was purified by FC (pentane/EtOAc 95:5), giving a mixture of lactone **22** and ester *E*-**21** (25 mg, **22**/*E*-**21** = 5:2 ratio, NMR data). This mixture was acetylated with acetic anhydride (0.2 mL, 2.6 mmol) and DMAP (0.5 mol%) in pyridine (5 mL) at room temperature for 48 h. The crude mixture was extracted with Et_2_O, neutralized with 10% aqueous H_2_SO_4_ and washed with brine. The organic layer was dried, and the solvent was removed under reduced pressure. The crude was purified by column chromatography (pentane/EtOAc 97:3) to afford pure lactone **22** (16 mg, 25%) and acetate **23** (8 mg, 10%).

Lactone **22**

White solid. M.p. 180–182 °C, from CH_2_Cl_2_. ${\left[\alpha \right]}_{D}^{20}$ = +11.4 (c = 2.83, CHCl_3_). IR (ν, cm^−1^): 3054, 2986, 2933, 2865, 1740, 1722. ^1^H NMR (400 MHz, CDCl_3_) δ_H_: 5.81 (dt, *J* = 5.4, 2.3 Hz, 1H, CH-17), 4.48 (d, *J* = 1.66 Hz, 1H, CH-15), 3.64 (s, 3H, -CO_2_CH_3_), 3.03 (m, 2H, CH_2_-18), 2.82 (bs, 1H, CH-13), 1.19 (s, 3H, CH_3_-20), 0.86 (s, 3H, CH_3_-22), 2.20–0.84 (m, 18H). ^13^C NMR (100 MHz, CDCl_3_) δ_C_: 177.9, 172.6, 148.9, 115.3, 88.0, 56.8, 54.6, 51.1, 46.1, 43.8, 40.8, 40.7, 39.6, 38.4, 38.0, 34.3, 33.1, 30.0, 28.7, 20.7, 19.0, 18.9, 15.7. GCMS *m*/*z* calculated for C_23_H_32_O_4_: 359.28; found 328.2 (M^+^ -CO_2_).

Ester **23**

Colorless oil. ${\left[a\right]}_{D}^{20}$= −69.8 (c = 2.36, CHCl_3_). IR (ν, cm^−1^): 2984, 2930, 2859, 1727. ^1^H NMR (400 MHz, CDCl_3_) δ_H_: 5.62 (t, *J* = 7.4 Hz, 1H, CH-17), 5.44 (s, 1H, CH-15), 4.12 (q, *J* = 7.8, 7.3 Hz, 2H, -CO_2_CH_2_CH_3_), 3.63 (s, 3H, -CO_2_CH_3_), 2.97 (d, *J* = 7.1 Hz, 2H, CH_2_-18), 2.79 (bs, 1H, CH-13), 2.07 (s, 3H, -O (CO)CH_3_), 1.25 (t, *J* = 7.9 Hz, 3H, -CO_2_CH_2_CH_3_), 1.15 (s, 3H, CH_3_-20), 0.81 (s, 3H, CH_3_-22), 2.20–0.80 (m, 18H). ^13^C NMR (100 MHz, CDCl_3_) δ_C_: 177.9, 171.5, 170.7, 148.7, 117.3, 80.7, 60.6, 56.6, 52.5, 51.1, 47.8, 43.8, 42.3, 40.6, 39.7, 37.9, 37.0, 34.7, 34.3, 33.1, 28.6, 20.9, 20.8, 19.1, 18.4, 15.5, 14.2.

Bu_6_Sn_2_-mediated ATRA to methyl 15α-hydroxy-*ent*-kaurenoate

Methyl 15α-hydroxy-*ent*-kaurenoate **20** (50 mg, 0.15 mmol) in 3.2 mL benzene was treated under N_2_ with ethyl iodoacetate (36 µL, 0.3 mmol), PhSO_2_N_3_ (83 mg, 0.45 mmol), Bu_6_Sn_2_ (115 μL, 0.23 mmol) and DTBHN (0.9 mg, 0.005 mmol). The reaction mixture was refluxed for 5 h. The crude product was purified by FC (pentane/EtOAc 95:5) and gave a mixture of compounds **22** and *E*-**21** (43 mg, **22**/*E*-**21** = 5:2, NMR data), along with unreacted starting material (5 mg, 10%).

Et_3_B-mediated ATRA to methyl 15α-hydroxy-*ent*-kaurenoate

Methyl 15α-hydroxy-*ent*-kaurenoate **20** (50 mg, 0.15 mmol) in 3.5 mL EtOH was treated under N_2_ with ethyl iodoacetate (36 μL, 0.3 mmol), PhSO_2_N_3_ (83 mg, 0.45 mmol) and Et_3_B (0.45 mL, 0.45 mmol) at room temperature overnight. The crude product was purified by column chromatography (pentane/EtOAc 95:5) and afforded unreacted starting material **20** (7 mg, 14%), ester *Z*-**21** (17 mg, 27%, contaminated with cca. 20% *E*-**21**), and pure ester *E*-**21** (15 mg, 24%).

Ester *E*-**21**

Colorless oil. ${\left[\alpha \right]}_{D}^{20}$ = −89.8 (c = 4.80, CHCl_3_). IR (ν, cm^−1^): 3484, 2933, 2871, 1724, 1463, 1447, 1370, 1329, 1233, 1191, 1153. ^1^H NMR (400 MHz, CDCl_3_) δ_H_: 5.80 (t, *J* = 7.3 Hz, 1H, CH-17), 4.13 (q, *J* = 7.1 Hz, 2H, -CO_2_CH_2_CH_3_), 3.77 (s, 1H, CH-15), 3.64 (s, 3H, -CO_2_CH_3_), 3.09 (d, *J* = 7.5 Hz, 2H, CH_2_-COOEt), 1.25 (t, *J* = 7.1 Hz, 3H, -CO_2_CH_2_CH_3_), 1.17 (s, 3H, CH_3_-20), 0.83 (s, 3H, CH_3_-22) 2.85–0.72 (m, 18H). ^13^C NMR (100 MHz, CDCl_3_) δ_C_: 178.11, 171.90, 153.54, 116.42, 83.18, 60.69, 56.96, 52.89, 51.16, 47.33, 43.82, 40.75, 39.60, 38.02, 36.09, 35.14, 35.09, 34.65, 30.56, 28.72, 21.03, 19.13, 18.66, 15.64, 14.19.

Ester *Z*-**21**

Colorless oil. IR (ν, cm^−1^): 3484, 2932, 2872, 1725, 1464, 1448, 1372, 1312, 1233, 1191, 1155. ^1^H NMR (400 MHz, CDCl_3_) selected values, δ_H_: 5.50 (t, *J* = 7.8 Hz, 1H, CH-17), 4.14 (q, *J* = 7.1 Hz, 2H, -CO_2_CH_2_CH_3_), 3.95 (s, 1H, CH-15), 3.64 (s, 3H, -CO_2_CH_3_), 3.11-3.32 (m, 2H, CH_2_-COOEt), 1.25 (t, *J* = 7.1 Hz, 3H, -CO_2_CH_2_CH_3_), 1.18 (s, 3H, CH_3_-20), 0.82 (s, 3H, CH_3_-22). ^13^C NMR (100 MHz, CDCl_3_) selected values, δ_C_: 178.03, 172.79, 154.79, 115.62, 80.40, 60.96, 57.06, 53.11, 51.05, 47.79, 43.84, 42.13, 40.80, 39.61, 38.06, 36.00, 35.12, 33.18, 28.69, 21.07, 19.15, 18.27, 15.57, 14.13.

Bu_6_Sn_2_-mediated ATRA of iodoacetic acid to methyl 15α-hydroxy-*ent*-kaurenoate

Methyl 15α-hydroxy-*ent*-kaurenoate **20** (64.0 mg, 0.192 mmol) in 2.0 mL benzene was treated under N_2_ with iodoacetic acid (71.4 mg, 0.38 mmol), PhSO_2_N_3_ (105.0 mg, 0.57 mmol), Bu_6_Sn_2_ (145 μL, 0.28 mmol), and DTBHN (1.0 mg, 0.006 mmol). The reaction mixture was refluxed for 5 h. The crude product was purified by CC (petroleum ether/EtOAc 70:30) to provide lactone **22** (23 mg, 28%) and azide **24** as a colorless oil (91 mg, 42%, contaminated with PhSO_2_N_3_).

Azide **24**

Colorless oil. IR (ν, cm^−1^): 3675, 2988, 2955, 2928, 2902, 2108, 1724, 1553. ^1^H NMR (400 MHz, D_3_COD) δ_H_: 3.63 (s, 3H, -CO_2_CH_3_), 3.52 (s, 1H, CH-15), 2.46 (t, 2H, CH_2_-17), 1.17 (s, 3H, CH_3_-20), 0.84 (s, 3H, CH_3_-22), 2.29–0.83 (m, 21H). ^13^C NMR (100 MHz, D_3_COD) δ_C_: 179.6 (2xC (O)2), 88.3, 76.0, 58.1, 56.3, 51.6, 48.5, 45.0, 43.0, 41.8, 40.8, 39.1, 37.1, 36.7, 29.1, 29.0, 28.0, 26.7, 22.3, 20.1, 19.6, 16.0.

Et_3_B-mediated ATRA of iodoacetonitrile to 15α-hydroxy-*ent*-kaurenoic acid

15α-Hydroxy-*ent*-kaurenoic acid **18** (70 mg, 0.22 mmol) in 2.0 mL EtOH was treated under N_2_ with iodoacetonitrile (32 μL, 0.44 mmol), PhSO_2_N_3_ (120 mg, 0.66 mmol), and Et_3_B (0.88 mL, 0.88 mmol). The reaction mixture was purified by FC (CH_2_Cl_2_/MeOH 95:5) and provided compound **25** (48 mg, 54%) as a colorless oil (contaminated with PhSO_2_N_3_).

Nitrile **25**

Colorless oil. IR (ν, cm^−1^): 3836, 3673, 2988, 2973, 2902, 2653, 1698, 1404, 1394, 1381, 1242. ^1^H NMR (400 MHz, D_3_COD) δ_H_: 5.29 (s, 1H, OH), 2.71–2.81 (m, 2H, CH_2_-18), 2.54 (s, 1H, CH-15), 1.24 (s, 3H, CH_3_-20), 0.94 (s, 3H, CH_3_-22), 2.42–0.77 (m, 21H). ^13^C NMR (100 MHz, D_3_COD) δ_H_: 183.9, 119.4, 85.5, 83.2, 56.4, 55.4, 53.4, 50.3, 47.5, 43.6, 40.5, 39.8, 38.8, 37.9, 37.6, 36.1, 28.8, 26.4, 20.8, 19.1, 18.8, 15.5.

Azidoester **26**

To a stirred solution of azidoacid **24** (45 mg, 0.10 mmol) in approximately 5 mL of Et_2_O at 0 °C, an etheric solution of CH_2_N_2_ was added dropwise until the yellow color persisted (0.2–0.25 mmol). The mixture was stirred for 30 min and concentrated to give the crude product, which was purified by column chromatography (pentane/EtOAc 95:5) to give azidoester **26** (41 mg, 99%) as a colorless oil.

Colorless oil. ${\left[\alpha \right]}_{D}^{20}$ = −55.8 (c = 2.13, CHCl_3_). IR (ν, cm^−1^): 3737, 3526 (w), 2940, 2872, 2853, 2299, 2107, 1725, 1445. ^1^H NMR (400 MHz, CDCl_3_) δ_H_: 3.69 (s, 3H, C-19 (-CO_2_CH_3_)), 3.63 (s, 3H, C-21, -CO_2_CH_3_)), 3.40 (s, 1H, CH-15), 2.56 (t, *J* = 7.9 Hz, 2H, CH_2_-18), 1.16 (s, 3H, CH_3_-20), 0.81 (s, 3H, CH_3_-22), 2.16–0.78 (m, 21H). ^13^C NMR (100 MHz, CDCl_3_) δ_C_: 177.84, 173.61, 85.22, 75.44, 56.71, 54.72, 51.71, 51.06, 47.55, 43.78, 41.87, 40.59, 39.59, 37.98, 36.05, 35.66, 29.41, 28.63, 28.41, 25.78, 21.03, 18.99, 18.19, 15.34.

Lactam **27**

A solution of azidoester **26** (53 mg, 0.12 mmol) and 10% Pd/C (10% *w*/*w*) in dry EtOAc (2 mL) was stirred for 64 h at room temperature under H_2_ (1 atm). The catalyst was filtered off, the solvent was removed under reduced pressure, and the crude purified by FC (CH_2_Cl_2_/MeOH 85:15), giving lactam **27** (16 mg, 35%) as a yellowish oil.

Yellowish oil. ${\left[\alpha \right]}_{D}^{20}$ = −90.8 (c = 3.0, CHCl_3_). IR (ν, cm^−1^): 3351, 2933, 2871, 2322, 1722, 1685, 1552. ^1^H NMR (400 MHz, CDCl_3_) δ_H_: 6.73 (s, 1H, NH), 3.63 (s, 3H, -CO_2_CH_3_), 3.49 (s, 1H, CH-15), 2.44–2.27 (m, 2H), 1.16 (s, 3H, CH_3_-20), 0.81 (s, 3H, CH_3_-22), 2.14–0.79 (m, 21H). ^13^C NMR (100 MHz, CDCl_3_) δ_C_: 179.1, 178.0, 88.2, 70.8, 56.8, 54.4, 51.1, 47.4, 44.9, 43.7, 40.7, 38.0, 39.6, 36.6, 35.4, 30.6, 29.3, 28.6, 26.3, 21.2, 19.1, 19.0, 15.5.

## 4. Conclusions

Modifications of *ent*-kaurenoic acid derivatives at the *exo*-methylenic double bond of the tetracyclic diterpenic framework based on mild radical mediated C–C bond forming reactions such as the iodine ATRA and the azidoalkylation reaction have been performed. Although this reaction has been demonstrated previously on other substrates, their application in complex natural compounds is not well documented and far from trivial due to the potential side reactions such as intramolecular hydrogen atom transfers. Both steric and electronic factors strongly influence the outcome of the free radical sequences, providing unprecedented avenues for expanding structural diversity of natural products.

The azidoalkylation reaction proved to be extremely useful in the context of increasing structural complexity of the parent natural scaffold, including simultaneous functionalization with different heteroatomic functional groups. The advantage of the tertiary azide moiety was demonstrated by subsequent reductions, leading to spiro-fused lactam and pyrrolidine fragments. The reported structural modifications led to a series of new compounds with relevant biological activity, which was demonstrated by in vitro cytotoxicity tests on several tumor cell lines. The hybrid terpene-nitrogen containing heterocycles with unprecedented spiro-junction have shown relevant cytotoxicity and promising selectivity indexes. These results represent a solid basis for following research on the synthesis of such derivatives based on available natural product templates.

## Figures and Tables

**Table 1:** Antiproliferative activity of *ent*-kauranic derivatives. DT–docetaxel; SP–staurosporine.

Compound	Conc. Unit	IC_50_							
		hTERT RPE-1	Capan-1	HCT-116	NCI-H460	DND-41	HL-60	K-562	Z-138
**7**	µM	29.2 ± 1.2	37.1 ± 3.9	30.7 ± 16.4	12.0 ± 1.5	36.5 ± 0.7	19.6 ± 7.7	24.7 ± 11.8	27.6 ± 15.5
**11**	µM	1.8 ± 0.4	1.7 ± 0.5	1.0 ± 0.4	1.3 ± 0.1	1.4 ± 0.3	3.0 ± 1.4	8.8 ± 2.2	0.5 ± 0.2
**12**	µM	7.6 ± 0.5	1.2 ± 0.7	0.8 ± 0.2	1.4 ± 0.1	21.4 ± 10.1	38.3 ± 17.8	36.4 ± 11.5	52.0 ± 33.4
**13**	µM	32.2 ± 2.0	11.5 ± 1.0	11.6 ± 0.5	1.0 ± 0.1	6.1 ± 1.1	8.3 ± 1.3	3.2 ± 1.0	9.5 ± 0.9
**14**	µM	26.2 ± 0.9	44.5 ± 8.3	5.8 ± 0.1	31.1 ± 1.6	44.5 ± 4.8	46.2 ± 9.1	41.0	22.7
**16**	µM	38.2 ± 5.1	18.1 ± 3.4	19.0 ± 5.7	15.4 ± 6.0	7.8 ± 1.0	33.6 ± 2.6	58.4 ± 2.5	22.1 ± 11.4
**22**	µM	1.4 ± 0.1	1.3 ± 0.6	0.5 ± 0.2	1.4 ± 0.3	2.3 ± 0.3	3.7 ± 1.3	2.5 ± 0.1	2.4 ± 1.1
**24**	µM	31.7 ± 3.1	33.2 ± 7.6	18.8 ± 8.0	41.8 ± 5.3	38.9 ± 4.1	49.3 ± 5.0	76.3 ± 1.4	25.2 ± 0.3
**25**	µM	33.6 ± 4.5	6.4 ± 1.5	34.4 ± 0.5	34.0 ± 2.9	36.4 ± 9.8	>100	>100	52.0 ± 17.4
**26**	µM	25.9 ± 0.3	8.3 ± 1.1	9.9 ± 2.7	0.6 ± 0.2	6.6 ± 1.3	41.1 ± 15.9	36.9 ± 0.2	21.6 ± 5.4
**27**	µM	27.7 ± 4.3	3.7 ± 1.7	4.0 ± 3.7	1.9 ± 0.2	8.3 ± 2.0	38.0 ± 11.8	56.5 ± 2.8	39.8 ± 10.9
**DT**	nM	18.7 ± 4.8	4.2 ± 1.8	2.2 ± 0.8	5.5 ± 1.3	4.7 ± 1.2	4.3 ± 1.6	5.2 ± 1.2	3.7 ± 0.7
**SP**	nM	1.0	6.2 ± 1.8	1.5	2.2 ± 0.8	8.6 ± 1.5	9.1 ± 1.6	27.9 ± 3.2	6.7 ± 4.4

## Data Availability

Data of the compounds are available on request from the corresponding authors.

## Supplementary Materials

The following are available online. X-ray crystal structure report for lactam **12** (CCDC 2023783), X-ray crystal structure report for lactone **22** (CCDC 2023785), IR and NMR spectra of compounds **11**, **12**, **13**, **16**, **22**, **25**, **26**, **27**, IC_50_, and SI data for investigated *ent*-kauranic derivatives.

## Sample Availability

Samples of the compounds are available on request from the corresponding authors.
