# Supplementary material for: Preparation of Antiproliferative Terpene-Alkaloid Hybrids by Free Radical-Mediated Modification of ent-Kauranic Derivatives
Source: Molecules. 2021 Jul 28;26(15):4549. doi: 10.3390/molecules26154549 (PMC8347134; doi:10.3390/molecules26154549)
Supplement: Supplementary file 1 [file molecules-26-04549-s001.zip › molecules-1288203_si_revised_2.pdf]

SUPPLEMENTARY MATERIAL

Preparation of Antiproliferative Terpene-Alkaloid Hybrids by Free Radical- Mediated Modification of *ent*-Kauranic Derivatives

Elena Pruteanu [1], Vladilena Gîrbu [1], Nicon Ungur [1], Leentje Persoons [2], Dirk Daelemans [2], Philippe Renaud [3,*], Veaceslav Kulciţki [1,*]

[1] Institute of Chemistry (MECC), str. Academiei, 3, MD-2028, Chișinău, Republic of Moldova; elena.pruteanu@unibe.ch (E.P.); vgirbu1@gmail.com (V.G.); nicon.ungur@gmail.com (N.U.)
[2] KU Leuven, Department of Microbiology, Immunology and Transplantation, Laboratory of Virology and Chemotherapy, Rega Institute for Medical Research, Herestraat 49, 3000 Leuven, Belgium; leentje.persoons@rega.kuleuven.be (L.P.); dirk.daelemans@kuleuven.be (D.D.)
[3] Department of Chemistry and Biochemistry, University of Bern, Freiestrasse 3, 3012 Bern, Switzerland; elena.pruteanu@unibe.ch (E.P.)
* Correspondence: philippe.renaud@unibe.ch (P.R.); kulcitki@yahoo.com (V.K.)

Content:

Supplementary data includes:

X-Ray crystal structure report for lactam **12** SI 2

X-Ray crystal structure report for lactone **22** SI 11

IR and NMR SPECTRA of compounds **11**, **12**, **13**, **16**, **22**, **25**, **26**, **27** SI 17

Table S14 with IC_{50} and SI data for investigated *ent*-kauranic derivatives SI 29

References SI 30

X-RAY CRYSTAL STRUCTURE REPORT

Lactam 12 (CCDC 2023783)

EXPERIMENTAL

Crystal-Structure Determination. A crystal of $C_{23}H_{35}NO_3$ was mounted in air at ambient conditions. All measurements were made on a *Oxford Diffraction SuperNova* area-detector diffractometer[1] using mirror optics monochromated Mo *K*α radiation (λ = 0.71073 Å) and Al filtered.[2] The unit cell constants and an orientation matrix for data collection were obtained from a least-squares refinement of the setting angles of reflections in the range 1.7° < θ < 27.6°. A total of 2905 frames were collected using ω scans, with 5+5 seconds exposure time, a rotation angle of 1.0° per frame, a crystal-detector distance of 65.0 mm, at T = 173(2) K.

Data reduction was performed using the *CrysAlisPro*[1] program. The intensities were corrected for Lorentz and polarization effects, and an absorption correction based on the multi-scan method using SCALE3 ABSPACK in *CrysAlisPro*[1] was applied. Data collection and refinement parameters are given in *Table 1.*

The structure was solved by direct methods using *SHELXT,*[3] which revealed the positions of all non-hydrogen atoms of the title compound. All but the two acidic H-atoms were placed in geometrically calculated positions and refined using a riding model where each H-atom was assigned a fixed isotropic displacement parameter with a value equal to 1.2Ueq of its parent atom (1.5U_{eq} for methyl groups). The positions of the two acidic H-atoms were let freely refined.
Refinement of the structure was carried out on F^2 using full-matrix least-squares procedures, which minimized the function $\Sigma w(F_o^2 - F_c^2)^2$. The weighting scheme was based on counting statistics and included a factor to downweight the intense reflections. All calculations were performed using the *SHELXL-2014/7*[4] program in OLEX2.[5]
The unit cell contains two symmetry independent molecules.

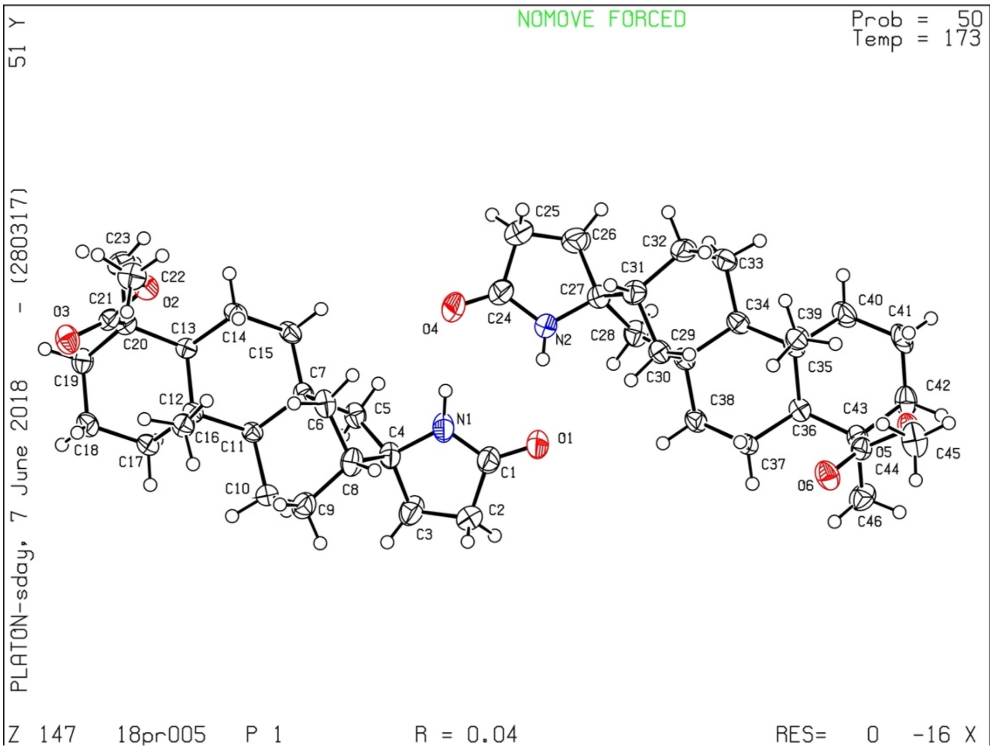

Table S1 Crystal data and structure refinement for 18PR005.

Identification code	18PR005
Empirical formula	$C_{23}H_{35}NO_3$
Formula weight	373.52
Temperature/K	172.99(10)
Crystal system	triclinic
Space group	P1
a/Å	6.9278(2)
b/Å	12.5083(3)
c/Å	13.0579(2)
α/°	68.573(2)
β/°	76.575(2)
γ/°	86.093(2)
Volume/Å^3	1024.37(4)
Z	2
ρ_{calc}g/cm^3	1.211
μ/mm^{-1}	0.079
F(000)	408.0
Crystal size/mm^3	0.416 × 0.334 × 0.23
Radiation	MoKα (λ = 0.71073)
2Θ range for data collection/°	3.438 to 56.492
Index ranges	$-9 \le h \le 9$, $-16 \le k \le 16$, $-17 \le l \le 16$
Reflections collected	44082
Independent reflections	9253 [R_{int} = 0.0343, R_{sigma} = 0.0276]
Data/restraints/parameters	9253/3/500
Goodness-of-fit on F^2	1.030

Final R indexes [I>=2σ (I)]	R_1 = 0.0429, wR_2 = 0.1023
Final R indexes [all data]	R_1 = 0.0477, wR_2 = 0.1065
Largest diff. peak/hole / e Å^{-3}	0.55/-0.30
Flack parameter	0.2(3)

Table S2 Fractional Atomic Coordinates (×10^4) and Equivalent Isotropic Displacement Parameters (Å^2×10^3) for 18PR005. U_{eq} is defined as 1/3 of of the trace of the orthogonalised U_{IJ} tensor.

Atom	*x*	*y*	*z*	U(eq)
O2	13370(3)	-189.4(16)	3827.6(15)	32.4(4)
O5	-2535(3)	9833.6(18)	9206.1(16)	36.9(4)
O3	15817(3)	-28.7(18)	2324.1(17)	39.3(4)
O6	145(3)	9857.2(19)	7870.6(18)	40.4(5)
O1	5113(4)	6528(2)	4741(2)	61.9(7)
N2	2525(4)	4541(2)	5982(2)	39.7(6)
O4	4853(5)	3277(2)	5593(3)	75.6(9)
C6	9262(3)	3366(2)	2824(2)	26.0(5)
C11	10711(3)	3548(2)	1677.7(19)	23.7(5)
N1	6904(4)	5308(2)	3968(2)	40.9(6)
C13	11152(3)	1404.1(19)	2205.9(19)	23.3(5)
C36	-2473(3)	8548(2)	7157(2)	26.8(5)
C20	12435(3)	368(2)	2063(2)	25.6(5)
C44	-1626(3)	9750(2)	8213(2)	27.5(5)
C21	14094(4)	52(2)	2723(2)	26.9(5)
C29	-555(3)	6509(2)	6694(2)	26.3(5)
C12	12261(3)	2591(2)	1616(2)	23.7(5)
C10	11581(4)	4792(2)	1167(2)	33.5(6)
C35	-2915(3)	7348(2)	8118(2)	27.2(5)
C14	9971(4)	1246(2)	3400(2)	27.5(5)
C39	-1736(4)	7162(2)	9029(2)	31.3(5)
C30	1201(4)	6074(2)	7274(2)	29.1(5)
C27	563(4)	4563(2)	6702(2)	34.7(6)
C7	10204(4)	3673(2)	3645(2)	31.3(5)
C9	11998(4)	5279(3)	2020(3)	40.0(7)
C19	13327(4)	631(2)	800(2)	31.3(5)
C34	-2538(4)	6410(2)	7565(2)	28.6(5)
C17	13055(4)	2755(2)	360(2)	31.3(5)
C43	-3056(3)	9585(2)	7551(2)	28.7(5)
C15	8376(4)	2153(2)	3332(2)	28.9(5)
C37	-386(4)	8614(2)	6401(2)	30.4(5)
C5	7626(4)	4289(2)	2629(2)	30.1(5)
C8	10370(4)	4973(2)	3114(2)	34.6(6)
C38	-228(4)	7730(2)	5834(2)	32.7(6)
C16	14018(4)	2650(2)	2121(2)	29.2(5)
C23	11116(4)	-722(2)	2499(3)	35.8(6)
C24	3191(6)	3496(3)	6053(3)	54.4(9)
C22	14778(4)	-584(3)	4540(2)	37.9(6)

C18	14392(4)	1792(2)	175(2)	34.4(6)
C28	-580(4)	5623(2)	6111(2)	33.8(6)
C31	835(4)	4777(2)	7754(2)	33.7(6)
C4	8245(4)	5293(2)	2918(2)	33.3(6)
C32	-1002(5)	4515(2)	8730(2)	40.0(6)
C45	-1216(4)	10021(3)	9836(3)	46.2(8)
C33	-2867(4)	5150(2)	8396(3)	39.7(7)
C46	-2853(5)	10718(3)	6516(3)	41.2(7)
C41	-5803(4)	8283(3)	9106(3)	43.4(7)
C1	6275(4)	6332(3)	3954(3)	46.5(8)
C42	-5245(4)	9442(3)	8190(3)	36.6(6)
C40	-5151(4)	7309(3)	8680(3)	41.0(7)
C3	7994(5)	6511(3)	2102(3)	46.0(7)
C26	-263(5)	3371(3)	6903(3)	49.6(8)
C25	1566(7)	2634(3)	6808(4)	68.4(12)
C2	7293(7)	7185(3)	2859(4)	70.3(13)

Table S3 Anisotropic Displacement Parameters ($Å^2×10^3$) for 18PR005. The Anisotropic displacement factor exponent takes the form: $-2\pi^2[h^2a^{*2}U_{11}+2hka^*b^*U_{12}+…]$.

Atom	U_{11}	U_{22}	U_{33}	U_{23}	U_{13}	U_{12}
O2	27.7(9)	39.7(10)	28.8(9)	-8.2(8)	-11.9(7)	4.0(7)
O5	26.7(9)	56.1(12)	35.9(10)	-25.4(10)	-8.2(8)	4.6(8)
O3	29.0(9)	48.6(12)	39.6(11)	-15.6(9)	-9.4(8)	9.8(8)
O6	24.9(9)	55.3(12)	45.2(11)	-24.7(10)	-4.1(8)	-2.1(8)
O1	55.3(14)	43.8(13)	79.7(18)	-35.9(13)	22.4(13)	-6.1(11)
N2	44.3(14)	30.1(12)	38.8(14)	-12.7(11)	1.9(11)	2.8(10)
O4	87(2)	37.2(13)	70.8(18)	-9.1(12)	20.2(15)	23.7(13)
C6	21.3(11)	31.9(12)	24.4(12)	-9.5(10)	-6.9(9)	5.6(9)
C11	24.0(11)	24.9(11)	20.4(11)	-5.6(9)	-5.7(9)	0.8(9)
N1	42.7(14)	44.2(15)	34.7(13)	-17.2(12)	-5.8(11)	14.9(11)
C13	22.1(11)	24.8(11)	22.3(11)	-6.3(9)	-7.6(9)	0.8(9)
C36	23.9(11)	32.7(13)	24.5(12)	-9.7(10)	-8.2(9)	2.6(9)
C20	25.0(11)	25.0(11)	28.0(12)	-9.4(10)	-8.5(9)	0.2(9)
C44	25.3(12)	27.5(12)	29.6(13)	-10.6(10)	-6.1(10)	3.7(9)
C21	27.7(12)	22.3(11)	31.5(13)	-9.7(10)	-8.6(10)	3.4(9)
C29	26.8(12)	31.4(13)	20.6(11)	-9.2(10)	-5.1(9)	0.2(9)
C12	21.5(10)	25.7(11)	22.9(11)	-8.0(9)	-4.6(9)	0.0(8)
C10	35.4(13)	25.6(12)	33.6(14)	-8.5(11)	0.0(11)	-0.5(10)
C35	21.4(11)	33.2(13)	25.0(12)	-10.4(10)	-0.5(9)	-3.6(9)
C14	24.6(11)	29.6(12)	22.9(12)	-3.2(10)	-5.0(9)	0.1(9)
C39	38.9(14)	31.2(13)	23.0(12)	-8.9(10)	-6.6(10)	1.6(10)
C30	26.2(11)	32.7(13)	28.6(13)	-11.1(11)	-6.2(10)	0.8(9)
C27	41.0(14)	30.9(13)	30.2(14)	-11.7(11)	-2.9(11)	-0.3(11)
C7	29.1(12)	43.4(15)	26.9(13)	-17.2(12)	-11.5(10)	8.5(10)
C9	33.2(14)	34.9(14)	55.3(18)	-21.9(14)	-7.0(13)	0.1(11)

C19	33.5(13)	34.6(13)	30.1(13)	-16.3(11)	-8.2(10)	3.7(10)
C34	24.4(11)	34.3(13)	27.2(13)	-11.2(11)	-4.6(10)	-3.7(10)
C17	34.6(13)	29.7(12)	23.1(12)	-6.4(10)	1.0(10)	-0.4(10)
C43	23.7(11)	34.7(13)	27.2(12)	-10.6(11)	-7.2(10)	5.7(9)
C15	21.0(11)	34.0(13)	25.7(12)	-5.7(10)	-2.5(9)	0.4(9)
C37	31.7(13)	29.8(13)	22.8(12)	-4.6(10)	-0.5(10)	-0.3(10)
C5	25.7(12)	37.0(13)	28.3(13)	-12.0(11)	-9.1(10)	7.4(10)
C8	36.3(14)	40.6(15)	36.4(14)	-22.4(12)	-13.7(11)	6.5(11)
C38	36.5(14)	34.4(14)	21.8(12)	-7.5(11)	-1.5(10)	3.6(11)
C16	20.9(10)	32.1(12)	37.2(14)	-14.8(11)	-7.2(10)	-0.2(9)
C23	37.6(14)	30.9(14)	41.7(15)	-12.5(12)	-14.1(12)	-2.9(11)
C24	72(2)	30.8(15)	45.6(18)	-9.0(14)	5.3(17)	7.8(15)
C22	36.3(14)	42.3(15)	35.6(15)	-8.5(12)	-19.2(12)	4.1(12)
C18	38.5(14)	37.2(14)	23.9(13)	-11.0(11)	-0.4(11)	2.5(11)
C28	39.2(14)	39.0(14)	27.7(13)	-15.5(11)	-10.7(11)	2.5(11)
C31	38.8(14)	31.4(14)	29.1(13)	-7.8(11)	-11.0(11)	6.2(11)
C4	33.9(13)	36.8(14)	29.8(13)	-14.0(11)	-7.5(11)	9.8(11)
C32	57.2(18)	29.7(13)	25.8(13)	-4.7(11)	-3.7(12)	0.4(12)
C45	36.6(15)	70(2)	46.8(18)	-34.4(17)	-17.4(14)	8.3(14)
C33	41.1(15)	36.1(15)	37.1(15)	-14.4(13)	5.8(12)	-11.3(12)
C46	50.1(17)	34.0(15)	39.9(16)	-11.4(13)	-16.5(13)	10.0(12)
C41	21.3(12)	58.1(19)	50.2(18)	-27.4(16)	6.9(12)	-2.7(12)
C1	37.1(15)	38.3(15)	65(2)	-28.5(15)	3.9(14)	-3.9(12)
C42	21.8(12)	49.9(17)	46.3(17)	-25.8(14)	-11.5(11)	8.3(11)
C40	24.9(13)	48.0(17)	48.4(17)	-21.4(14)	4.7(12)	-9.1(11)
C3	61(2)	37.4(16)	41.2(16)	-16.3(13)	-15.4(14)	16.0(14)
C26	64(2)	38.7(16)	44.2(18)	-17.6(14)	-1.3(15)	-10.6(14)
C25	90(3)	32.4(17)	63(2)	-12.2(17)	13(2)	-0.9(17)
C2	75(3)	32.2(17)	80(3)	-18.7(18)	26(2)	-5.6(17)

Table S4 Bond Lengths for 18PR005.

Atom	Atom	Length/Å	Atom	Atom	Length/Å
O2	C21	1.338(3)	C29	C28	1.560(4)
O2	C22	1.443(3)	C12	C17	1.544(3)
O5	C44	1.342(3)	C12	C16	1.530(3)
O5	C45	1.444(3)	C10	C9	1.536(4)
O3	C21	1.200(3)	C35	C39	1.537(3)
O6	C44	1.203(3)	C35	C34	1.567(4)
O1	C1	1.240(4)	C35	C40	1.549(3)
N2	C27	1.467(4)	C14	C15	1.522(3)
N2	C24	1.334(4)	C30	C31	1.526(4)
O4	C24	1.232(4)	C27	C28	1.541(4)
C6	C11	1.544(3)	C27	C31	1.545(4)
C6	C7	1.543(3)	C27	C26	1.542(4)
C6	C15	1.525(4)	C7	C8	1.517(4)

C6	C5	1.557(3)	C9	C8	1.540(4)
C11	C12	1.564(3)	C19	C18	1.522(4)
C11	C10	1.554(3)	C34	C33	1.549(4)
N1	C4	1.473(4)	C17	C18	1.525(4)
N1	C1	1.320(4)	C43	C46	1.547(4)
C13	C20	1.564(3)	C43	C42	1.539(3)
C13	C12	1.560(3)	C37	C38	1.525(4)
C13	C14	1.531(3)	C5	C4	1.548(4)
C36	C35	1.559(3)	C8	C4	1.553(4)
C36	C43	1.559(4)	C24	C25	1.508(5)
C36	C37	1.538(3)	C31	C32	1.533(4)
C20	C21	1.539(3)	C4	C3	1.533(4)
C20	C19	1.539(4)	C32	C33	1.536(4)
C20	C23	1.543(3)	C41	C42	1.509(5)
C44	C43	1.525(3)	C41	C40	1.517(4)
C29	C30	1.544(3)	C1	C2	1.483(5)
C29	C34	1.546(3)	C3	C2	1.501(5)
C29	C38	1.524(4)	C26	C25	1.524(5)

Table S5 Bond Angles for 18PR005.

Atom	Atom	Atom	Angle/°	Atom	Atom	Atom	Angle/°
C21	O2	C22	116.2(2)	N2	C27	C28	110.2(2)
C44	O5	C45	114.7(2)	N2	C27	C31	108.8(2)
C24	N2	C27	115.1(3)	N2	C27	C26	100.3(2)
C11	C6	C5	108.21(19)	C28	C27	C31	103.3(2)
C7	C6	C11	112.8(2)	C28	C27	C26	117.5(3)
C7	C6	C5	100.84(19)	C26	C27	C31	116.6(2)
C15	C6	C11	110.2(2)	C8	C7	C6	102.2(2)
C15	C6	C7	112.7(2)	C10	C9	C8	113.5(2)
C15	C6	C5	111.57(19)	C18	C19	C20	114.9(2)
C6	C11	C12	117.55(19)	C29	C34	C35	116.53(19)
C6	C11	C10	109.50(19)	C29	C34	C33	109.0(2)
C10	C11	C12	115.0(2)	C33	C34	C35	115.7(2)
C1	N1	C4	115.3(3)	C18	C17	C12	113.7(2)
C12	C13	C20	114.31(18)	C44	C43	C36	111.71(19)
C14	C13	C20	116.49(19)	C44	C43	C46	104.2(2)
C14	C13	C12	111.93(19)	C44	C43	C42	114.1(2)
C35	C36	C43	114.5(2)	C46	C43	C36	110.1(2)
C37	C36	C35	111.63(19)	C42	C43	C36	109.0(2)
C37	C36	C43	115.9(2)	C42	C43	C46	107.6(2)
C21	C20	C13	114.44(19)	C14	C15	C6	111.99(19)
C21	C20	C23	105.8(2)	C38	C37	C36	109.7(2)
C19	C20	C13	109.06(19)	C4	C5	C6	107.4(2)
C19	C20	C21	109.9(2)	C7	C8	C9	107.6(2)
C19	C20	C23	107.4(2)	C7	C8	C4	101.6(2)

C23	C20	C13	110.1(2)	C9	C8	C4	114.0(2)
O5	C44	C43	113.6(2)	C29	C38	C37	111.9(2)
O6	C44	O5	122.0(2)	N2	C24	C25	108.0(3)
O6	C44	C43	124.3(2)	O4	C24	N2	125.8(3)
O2	C21	C20	111.5(2)	O4	C24	C25	126.2(3)
O3	C21	O2	123.1(2)	C19	C18	C17	110.8(2)
O3	C21	C20	125.3(2)	C27	C28	C29	107.5(2)
C30	C29	C34	111.7(2)	C30	C31	C27	101.1(2)
C30	C29	C28	101.0(2)	C30	C31	C32	107.7(2)
C34	C29	C28	109.1(2)	C32	C31	C27	115.1(2)
C38	C29	C30	113.0(2)	N1	C4	C5	109.2(2)
C38	C29	C34	110.3(2)	N1	C4	C8	110.0(2)
C38	C29	C28	111.4(2)	N1	C4	C3	101.1(2)
C13	C12	C11	108.09(18)	C5	C4	C8	103.4(2)
C17	C12	C11	107.85(19)	C3	C4	C5	116.7(2)
C17	C12	C13	107.39(19)	C3	C4	C8	116.3(3)
C16	C12	C11	112.65(19)	C31	C32	C33	113.9(2)
C16	C12	C13	112.14(19)	C32	C33	C34	116.0(2)
C16	C12	C17	108.5(2)	C42	C41	C40	111.8(2)
C9	C10	C11	115.6(2)	O1	C1	N1	125.4(3)
C36	C35	C34	108.12(19)	O1	C1	C2	127.3(3)
C39	C35	C36	112.28(19)	N1	C1	C2	107.2(3)
C39	C35	C34	113.4(2)	C41	C42	C43	115.1(2)
C39	C35	C40	107.9(2)	C41	C40	C35	114.2(2)
C40	C35	C36	106.9(2)	C2	C3	C4	104.1(3)
C40	C35	C34	108.1(2)	C25	C26	C27	104.7(3)
C15	C14	C13	109.09(19)	C24	C25	C26	103.6(3)
C31	C30	C29	101.8(2)	C1	C2	C3	105.2(3)

Table S6 Hydrogen Atom Coordinates (Å×10[4]) and Isotropic Displacement Parameters (Å[2]×10[3]) for 18PR005.

Atom	*x*	*y*	*z*	U(eq)
H11	9857.73	3534.42	1179.62	28
H13	10131.69	1459.56	1780.61	28
H36	-3375.53	8585.03	6668.36	32
H10A	12810.79	4811.79	622.8	40
H10B	10661.71	5294.49	760.53	40
H14A	9360.55	484.89	3757.87	33
H14B	10851.39	1321.49	3849.48	33
H39A	-371.72	7381.29	8676.23	47
H39B	-1818.36	6365.85	9508.14	47
H39C	-2280.88	7621.6	9473.05	47
H30A	2464.78	6281.76	6734.83	35
H30B	1162.92	6372.39	7867.43	35
H7A	9357.28	3426.55	4398.25	38

H7B	11497.21	3331.3	3681.43	38
H9A	12130.56	6107.93	1667.07	48
H9B	13252.79	4986.71	2209.38	48
H19A	14257.2	34.56	725.09	38
H19B	12268.61	598.27	439.05	38
H34	-3563.22	6533.84	7129.37	34
H17A	11937.99	2805.58	14.62	38
H17B	13793.46	3476.99	-19.03	38
H15A	7576.66	2026.58	4083.39	35
H15B	7511.4	2074.62	2874.63	35
H37A	-134.68	9378.23	5830.54	37
H37B	602.47	8465.14	6852.57	37
H5A	7507.72	4559.87	1849.52	36
H5B	6354.34	3964.7	3110.74	36
H8	10682.98	5303.25	3632.27	41
H38A	-1207.76	7891.11	5376.3	39
H38B	1075.23	7795.47	5338.9	39
H16A	14536.63	3422.88	1813.06	44
H16B	15034.66	2145.38	1940.28	44
H16C	13583.23	2419.14	2925.48	44
H23A	10685.88	-972.55	3305.52	54
H23B	11862.07	-1318.22	2296.21	54
H23C	9979.59	-553.92	2167.08	54
H22A	15845.12	-31.22	4278.26	57
H22B	15296.68	-1307.58	4513.66	57
H22C	14129.11	-676.85	5302.49	57
H18A	14776.54	1924.33	-625.36	41
H18B	15587.7	1789.38	441.91	41
H28A	-1935.58	5406.82	6180.5	41
H28B	49.75	5954.89	5315.64	41
H31	1991.6	4371.48	8013.63	40
H32A	-707.33	4720.32	9326.32	48
H32B	-1279.95	3694.49	9031.75	48
H45A	-508.03	10737.37	9410.17	69
H45B	-287.83	9406.65	9972.85	69
H45C	-1974.73	10043.81	10542.91	69
H33A	-3803.91	5142.9	9078.04	48
H33B	-3474.43	4724.06	8060.24	48
H46A	-1482.37	10859.42	6130.91	62
H46B	-3335.73	11341.29	6762.25	62
H46C	-3614.35	10655.48	6011.91	62
H41A	-7231.23	8234.4	9389.74	52
H41B	-5190.73	8202.94	9726.2	52
H42A	-5542.36	10026.34	8525.2	44
H42B	-6070.88	9577.43	7649.61	44
H40A	-5915.5	7329.21	8138.27	49

H40B	-5456.08	6585.3	9309.36	49
H3A	9244.36	6820.54	1583.77	55
H3B	7021.38	6518.06	1672.17	55
H26A	-1133.34	3056.86	7646.1	60
H26B	-995.56	3418.43	6339.67	60
H25A	1861.27	2224.45	7541.52	82
H25B	1382.7	2083.81	6471.61	82
H2A	8405.48	7546.39	2948.99	84
H2B	6383.1	7775.22	2551.69	84
H2	3310(70)	5090(40)	5650(40)	84
H1	6450(60)	4670(40)	4540(40)	65(12)

X-RAY CRYSTAL STRUCTURE REPORT

Lactone 22 (CCDC 2023783)

EXPERIMENTAL

Crystal-Structure Determination. A crystal of $[C_{23}H_{32}O_4][H_2O]_{0.18}$ was mounted in air at ambient conditions. All measurements were made on a *Oxford Diffraction SuperNova* area-detector diffractometer[1] using mirror optics monochromated Mo *K*α radiation (λ = 0.71073 Å) and Al filtered.[2] The unit cell constants and an orientation matrix for data collection were obtained from a least-squares refinement of the setting angles of reflections in the range 2.2 < θ < 27.9°. A total of 1411 frames were collected using ω scans, with 5+5 seconds exposure time, a rotation angle of 1.0° per frame, a crystal-detector distance of 65.0 mm, at T = 173(2) K.
Data reduction was performed using the *CrysAlisPro*[1] program. The intensities were corrected for Lorentz and polarization effects, and an absorption correction based on the multi-scan method using SCALE3 ABSPACK in *CrysAlisPro*[1] was applied. Data collection and refinement parameters are given in *Table 1.*
The structure was solved by direct methods using *SHELXT*,[3] which revealed the positions of all non-hydrogen atoms of the title compound. The non-hydrogen atoms were refined anisotropically. All H-atoms were placed in geometrically calculated positions and refined using a riding model where each H-atom was assigned a fixed isotropic displacement parameter with a value equal to 1.2Ueq of its parent atom (1.5U_{eq} for the methyl groups and water).
Refinement of the structure was carried out on F^2 using full-matrix least-squares procedures, which minimized the function $\Sigma w(F_o^2 – F_c^2)^2$. The weighting scheme was based on counting statistics and included a factor to downweight the intense reflections. All calculations were performed using the *SHELXL-2014/7*[4] program in OLEX2.[5]
There is a site containing co-crystallized water which is only partially occupied. The partial occupation is associated with a slight conformational disorder of the ring O1-C1-C2-C3-C4-C5 which in some conformations leaves space in the crystal packing for a water molecule but not in others.

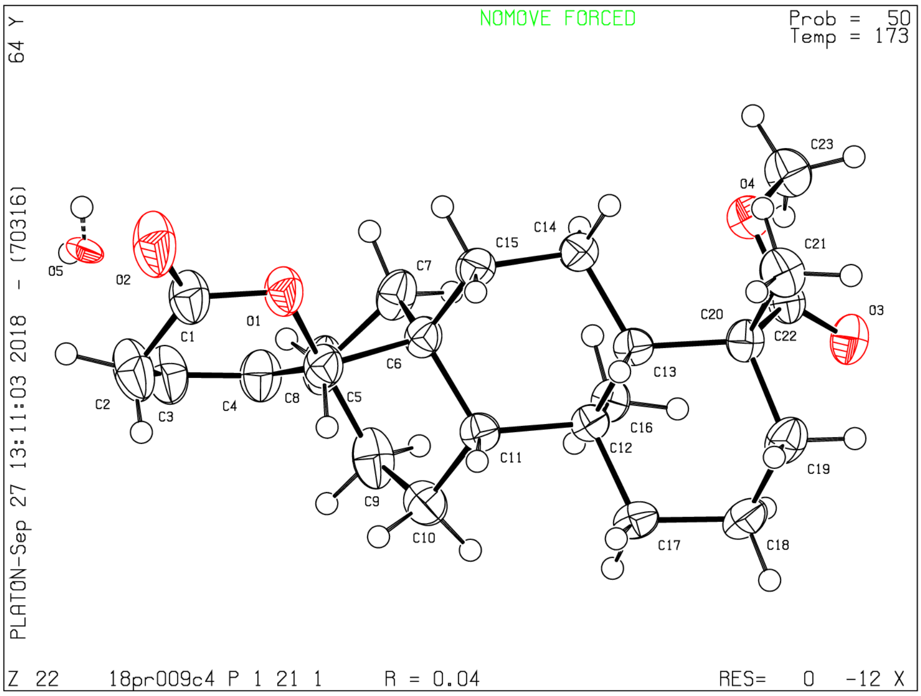

Table S7 Crystal data and structure refinement for 18PR009C4.

Identification code	18PR009C4
Empirical formula	$C_{23}H_{32.37}O_{4.19}$
Formula weight	375.82
Temperature/K	173.00(10)
Crystal system	monoclinic
Space group	$P2_1$
a/Å	7.1796(3)
b/Å	7.4191(3)
c/Å	18.9600(6)
α/°	90
β/°	98.674(4)
γ/°	90
Volume/Å^3	998.36(7)
Z	2
ρ_{calc}g/cm^3	1.250
μ/mm^{-1}	0.084
F(000)	408.0
Crystal size/mm^3	0.566 × 0.476 × 0.144
Radiation	MoKα (λ = 0.71073)
2Θ range for data collection/°	4.346 to 56.398
Index ranges	$-9 \le h \le 9$, $-9 \le k \le 9$, $-25 \le l \le 24$
Reflections collected	21285
Independent reflections	4577 [R_{int} = 0.0543, R_{sigma} = 0.0422]
Data/restraints/parameters	4577/5/263
Goodness-of-fit on F^2	1.024
Final R indexes [I>=2σ (I)]	R_1 = 0.0435, wR_2 = 0.0919

Final R indexes [all data]	$R_1 = 0.0561$, $wR_2 = 0.0999$
Largest diff. peak/hole / e Å^{-3}	0.17/-0.16
Flack parameter	-0.6(6)

Table S8. Fractional Atomic Coordinates (×10^4) and Equivalent Isotropic Displacement Parameters (Å^2×10^3) for 18PR009C4. U_{eq} is defined as 1/3 of of the trace of the orthogonalised U_{IJ} tensor.

Atom	*x*	*y*	*z*	U(eq)
O(1)	3436(3)	5016(3)	4643.9(9)	41.0(5)
O(2)	4076(3)	5513(4)	5802.3(10)	63.0(7)
O(3)	2619(2)	5976(3)	162.5(9)	40.6(5)
O(4)	2167(2)	7870(2)	1038.1(9)	35.4(4)
C(1)	3321(4)	4579(5)	5327.1(14)	45.4(7)
C(2)	2217(5)	2932(5)	5445.2(15)	58.1(9)
C(3)	484(5)	2808(5)	4890.0(15)	53.1(8)
C(4)	732(4)	3128(4)	4226.4(14)	43.2(7)
C(5)	2672(4)	3693(4)	4097.6(12)	35.1(6)
C(6)	2385(3)	4548(4)	3341.1(12)	31.3(6)
C(7)	265(4)	5057(4)	3251.0(14)	40.8(7)
C(8)	-618(4)	3419(5)	3547.8(14)	46.4(8)
C(9)	-585(4)	1858(5)	3022.5(15)	48.6(8)
C(10)	1436(4)	1379(4)	2902.2(14)	40.0(6)
C(11)	2757(3)	3003(3)	2825.3(12)	29.9(5)
C(12)	2892(3)	3562(3)	2032.3(12)	28.2(5)
C(13)	4191(3)	5241(3)	2046.0(12)	28.8(5)
C(14)	3562(4)	6766(4)	2501.8(13)	33.5(6)
C(15)	3677(4)	6137(4)	3273.7(13)	34.8(6)
C(16)	947(3)	3876(4)	1584.0(12)	32.4(5)
C(17)	3874(4)	1991(4)	1698.4(14)	35.5(6)
C(18)	4358(4)	2433(4)	961.1(14)	40.1(7)
C(19)	5549(4)	4133(4)	975.8(14)	40.2(7)
C(20)	4698(3)	5780(4)	1298.9(13)	30.9(5)
C(21)	6185(4)	7306(4)	1377.9(15)	40.8(7)
C(22)	3049(3)	6493(4)	766.7(13)	31.5(5)
C(23)	656(4)	8699(4)	555.2(15)	41.8(7)
O(5)	-1528(13)	4671(11)	5367(5)	27(3)

Table S9 Anisotropic Displacement Parameters (Å^2×10^3) for 18PR009C4. The Anisotropic displacement factor exponent takes the form: -$2\pi^2[h^2a^{*2}U_{11}+2hka^*b^*U_{12}+...]$.

Atom	U_{11}	U_{22}	U_{33}	U_{23}	U_{13}	U_{12}
O(1)	47.8(11)	48.6(12)	25.6(8)	1.1(8)	1.8(8)	-6.3(10)
O(2)	75.3(15)	82.0(18)	29.8(10)	-0.7(11)	1.3(10)	-30.0(14)
O(3)	42.0(10)	52.4(12)	27.2(9)	-1.4(8)	5.1(8)	-2.3(9)

O(4)	33.1(9)	38.9(11)	31.8(8)	1.6(8)	-3.2(7)	5.2(8)
C(1)	48.3(16)	57.2(19)	29.2(13)	4.0(13)	0.6(12)	-7.2(15)
C(2)	71(2)	70(2)	30.7(14)	10.3(15)	-1.9(14)	-24.3(19)
C(3)	57.0(19)	64(2)	38.0(15)	3.9(15)	7.8(13)	-20.9(18)
C(4)	40.6(15)	54.6(18)	33.9(13)	1.5(13)	3.9(12)	-4.2(14)
C(5)	37.1(14)	40.0(15)	26.8(12)	1.2(11)	0.3(10)	1.9(12)
C(6)	31.9(13)	36.7(14)	24.1(11)	1.1(10)	0.8(10)	5.2(11)
C(7)	35.0(14)	57.4(19)	30.2(12)	1.6(13)	5.3(11)	13.0(14)
C(8)	31.1(14)	74(2)	34.2(14)	4.0(15)	6.7(11)	3.5(15)
C(9)	36.4(15)	71(2)	37.2(14)	7.7(15)	1.7(12)	-15.1(15)
C(10)	44.6(15)	38.0(16)	35.6(13)	1.9(12)	0.6(11)	-6.1(13)
C(11)	28.2(12)	31.0(13)	29.1(12)	1.3(11)	-0.6(10)	3.3(11)
C(12)	27.5(12)	28.9(13)	27.7(11)	-1.4(10)	2.2(9)	1.9(10)
C(13)	26.3(12)	29.1(13)	29.8(11)	-0.1(10)	0.1(9)	2.3(10)
C(14)	40.0(14)	30.8(13)	29.9(12)	-0.7(11)	5.6(11)	1.4(12)
C(15)	45.4(15)	31.3(14)	26.8(11)	-2.2(11)	2.3(11)	1.5(12)
C(16)	29.6(12)	38.4(14)	28.4(11)	-0.9(11)	1.9(10)	-0.3(11)
C(17)	35.5(14)	31.7(14)	38.8(13)	-4.8(11)	4.1(11)	5.7(12)
C(18)	43.1(15)	40.0(16)	38.3(14)	-9.0(12)	10.0(12)	7.5(13)
C(19)	32.4(14)	49.5(18)	40.7(14)	-2.7(13)	11.8(11)	7.8(13)
C(20)	24.7(11)	37.3(14)	31.0(12)	1.0(11)	5.1(10)	-0.1(11)
C(21)	29.2(13)	47.2(17)	45.5(15)	3.4(13)	4.4(12)	-6.1(12)
C(22)	27.4(12)	36.0(14)	31.6(12)	2.4(11)	6.3(10)	-3.2(11)
C(23)	34.6(14)	47.5(17)	40.5(14)	7.5(13)	-3.2(12)	5.5(13)
O(5)	37(6)	12(5)	36(5)	6(4)	12(4)	5(4)

Table S10 Bond Lengths for 18PR009C4.

**Atom**	**Atom**	**Length/Å**	**Atom**	**Atom**	**Length/Å**
O(1)	C(1)	1.350(3)	C(8)	C(9)	1.530(5)
O(1)	C(5)	1.471(3)	C(9)	C(10)	1.544(4)
O(2)	C(1)	1.199(3)	C(10)	C(11)	1.554(4)
O(3)	C(22)	1.203(3)	C(11)	C(12)	1.577(3)
O(4)	C(22)	1.345(3)	C(12)	C(13)	1.554(3)
O(4)	C(23)	1.447(3)	C(12)	C(16)	1.539(3)
C(1)	C(2)	1.492(4)	C(12)	C(17)	1.546(3)
C(2)	C(3)	1.506(4)	C(13)	C(14)	1.533(3)
C(3)	C(4)	1.318(4)	C(13)	C(20)	1.567(3)
C(4)	C(5)	1.509(4)	C(14)	C(15)	1.526(3)
C(4)	C(8)	1.505(4)	C(17)	C(18)	1.526(4)
C(5)	C(6)	1.553(3)	C(18)	C(19)	1.522(4)
C(6)	C(7)	1.553(3)	C(19)	C(20)	1.534(4)
C(6)	C(11)	1.556(3)	C(20)	C(21)	1.548(4)
C(6)	C(15)	1.517(4)	C(20)	C(22)	1.529(3)
C(7)	C(8)	1.517(5)			

Table S11 Bond Angles for 18PR009C4.

Atom	Atom	Atom	Angle/°	Atom	Atom	Atom	Angle/°
C(1)	O(1)	C(5)	116.4(2)	C(10)	C(11)	C(6)	110.32(19)
C(22)	O(4)	C(23)	115.6(2)	C(10)	C(11)	C(12)	114.8(2)
O(1)	C(1)	C(2)	116.8(3)	C(13)	C(12)	C(11)	108.53(19)
O(2)	C(1)	O(1)	119.7(3)	C(16)	C(12)	C(11)	112.67(19)
O(2)	C(1)	C(2)	123.4(3)	C(16)	C(12)	C(13)	112.4(2)
C(1)	C(2)	C(3)	110.3(3)	C(16)	C(12)	C(17)	108.7(2)
C(4)	C(3)	C(2)	116.1(3)	C(17)	C(12)	C(11)	106.8(2)
C(3)	C(4)	C(5)	117.7(3)	C(17)	C(12)	C(13)	107.54(18)
C(3)	C(4)	C(8)	132.8(3)	C(12)	C(13)	C(20)	114.23(19)
C(8)	C(4)	C(5)	108.2(2)	C(14)	C(13)	C(12)	111.97(19)
O(1)	C(5)	C(4)	109.1(2)	C(14)	C(13)	C(20)	116.7(2)
O(1)	C(5)	C(6)	111.2(2)	C(15)	C(14)	C(13)	109.7(2)
C(4)	C(5)	C(6)	105.4(2)	C(6)	C(15)	C(14)	111.9(2)
C(5)	C(6)	C(11)	105.6(2)	C(18)	C(17)	C(12)	113.2(2)
C(7)	C(6)	C(5)	101.06(19)	C(19)	C(18)	C(17)	111.5(2)
C(7)	C(6)	C(11)	111.6(2)	C(18)	C(19)	C(20)	114.3(2)
C(15)	C(6)	C(5)	113.4(2)	C(19)	C(20)	C(13)	108.6(2)
C(15)	C(6)	C(7)	113.8(2)	C(19)	C(20)	C(21)	108.1(2)
C(15)	C(6)	C(11)	110.76(19)	C(21)	C(20)	C(13)	110.5(2)
C(8)	C(7)	C(6)	102.8(2)	C(22)	C(20)	C(13)	114.98(18)
C(4)	C(8)	C(7)	100.4(2)	C(22)	C(20)	C(19)	109.1(2)
C(4)	C(8)	C(9)	112.2(3)	C(22)	C(20)	C(21)	105.4(2)
C(7)	C(8)	C(9)	108.5(2)	O(3)	C(22)	O(4)	122.6(2)
C(8)	C(9)	C(10)	112.2(2)	O(3)	C(22)	C(20)	125.4(2)
C(9)	C(10)	C(11)	115.8(2)	O(4)	C(22)	C(20)	111.9(2)
C(6)	C(11)	C(12)	116.5(2)				

Table S12 Hydrogen Atom Coordinates (Å×10[4]) and Isotropic Displacement Parameters (Å[2]×10[3]) for 18PR009C4.

Atom	*x*	*y*	*z*	U(eq)
H(2A)	1849.26	2975.9	5916.65	70
H(2B)	2992.39	1870.65	5419.61	70
H(3)	-691.58	2522.88	5008.37	64
H(5)	3504.55	2643.04	4113.32	42
H(7A)	66.35	6133.45	3520.22	49
H(7B)	-243.47	5248.83	2753.05	49
H(8)	-1896.69	3670.66	3642.69	56
H(9A)	-1326.34	2182.1	2569.46	58
H(9B)	-1162.26	806.63	3203.96	58
H(10A)	1357.45	648.15	2474.48	48
H(10B)	2011.59	643.86	3299.36	48

H(11)	4020.86	2563.48	3015.15	36
H(13)	5391.82	4837.58	2315.15	35
H(14A)	4366.44	7809.3	2480.49	40
H(14B)	2278.31	7113.55	2317.39	40
H(15A)	4964.3	5795.69	3454.5	42
H(15B)	3333.06	7125.97	3563.01	42
H(16A)	128.87	2887.32	1651.23	49
H(16B)	1080.88	3960.6	1088.94	49
H(16C)	417.15	4975.76	1732.31	49
H(17A)	5022.91	1681.93	2013.23	43
H(17B)	3055.43	944.48	1661.36	43
H(18A)	5040.8	1430.4	793.89	48
H(18B)	3202.91	2596.2	629.07	48
H(19A)	5729.81	4421.69	491.73	48
H(19B)	6779.15	3891.62	1247.76	48
H(21A)	6591.72	7520.97	925.52	61
H(21B)	7246.58	6956.15	1721.02	61
H(21C)	5640.21	8386.73	1536.02	61
H(23A)	1162.74	9266.06	170.46	63
H(23B)	41.25	9586.98	807.58	63
H(23C)	-238.31	7793.48	367.14	63
H(5A)	-870(120)	5520(130)	5570(70)	41
H(5B)	-2700(40)	4470(170)	5280(70)	41

Table S13 Atomic Occupancy for 18PR009C4.

**Atom**	***Occupancy***	**Atom**	***Occupancy***	**Atom**	***Occupancy***
O(5)	0.184(6)	H(5A)	0.184(6)	H(5B)	0.184(6)

IR AND NMR SPECTRA

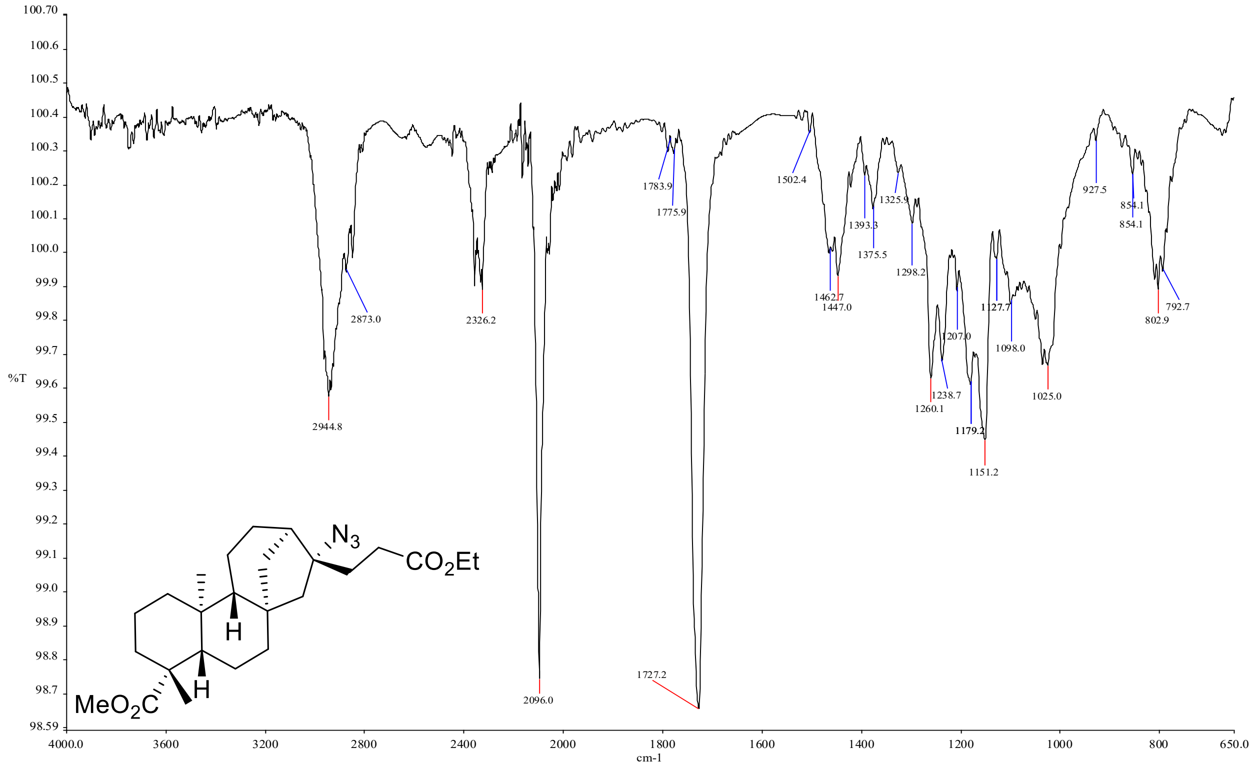

IR spectrum of azide **11**.

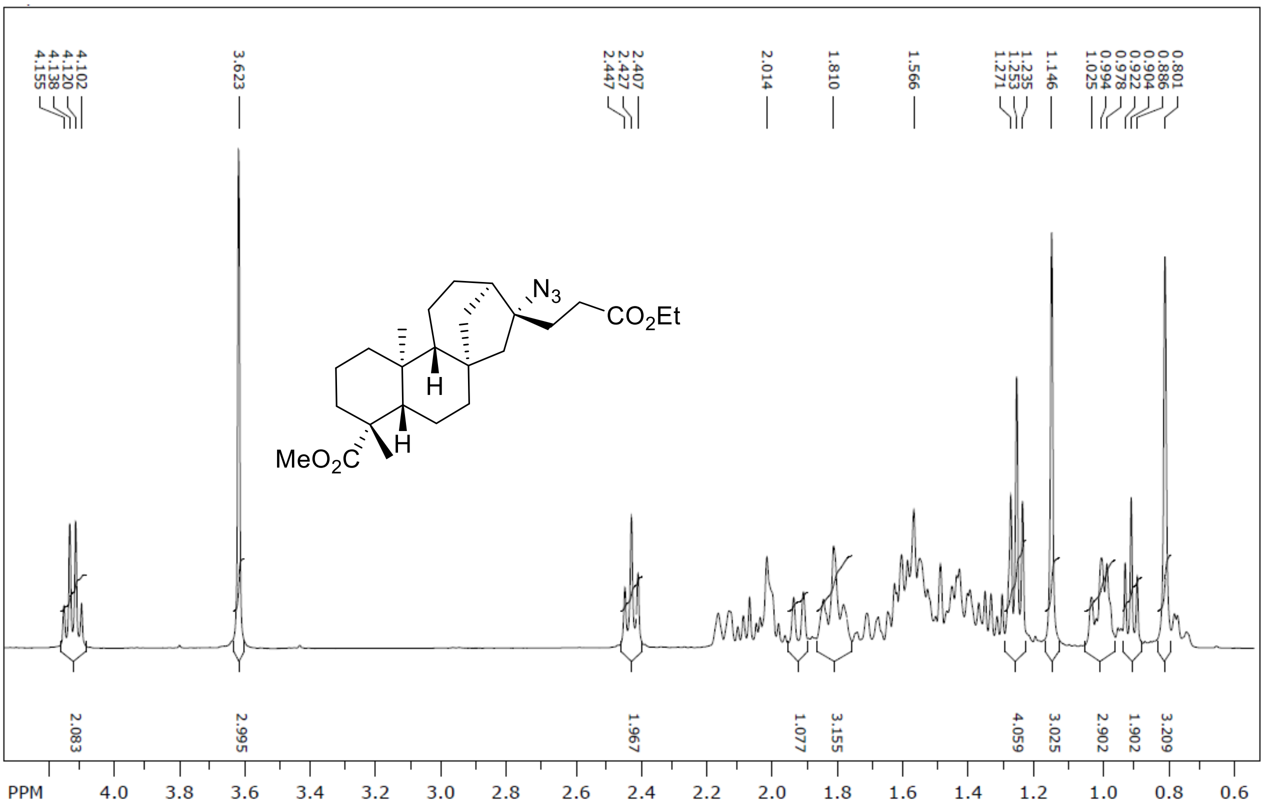

^{1}H NMR spectrum of azide **11**.

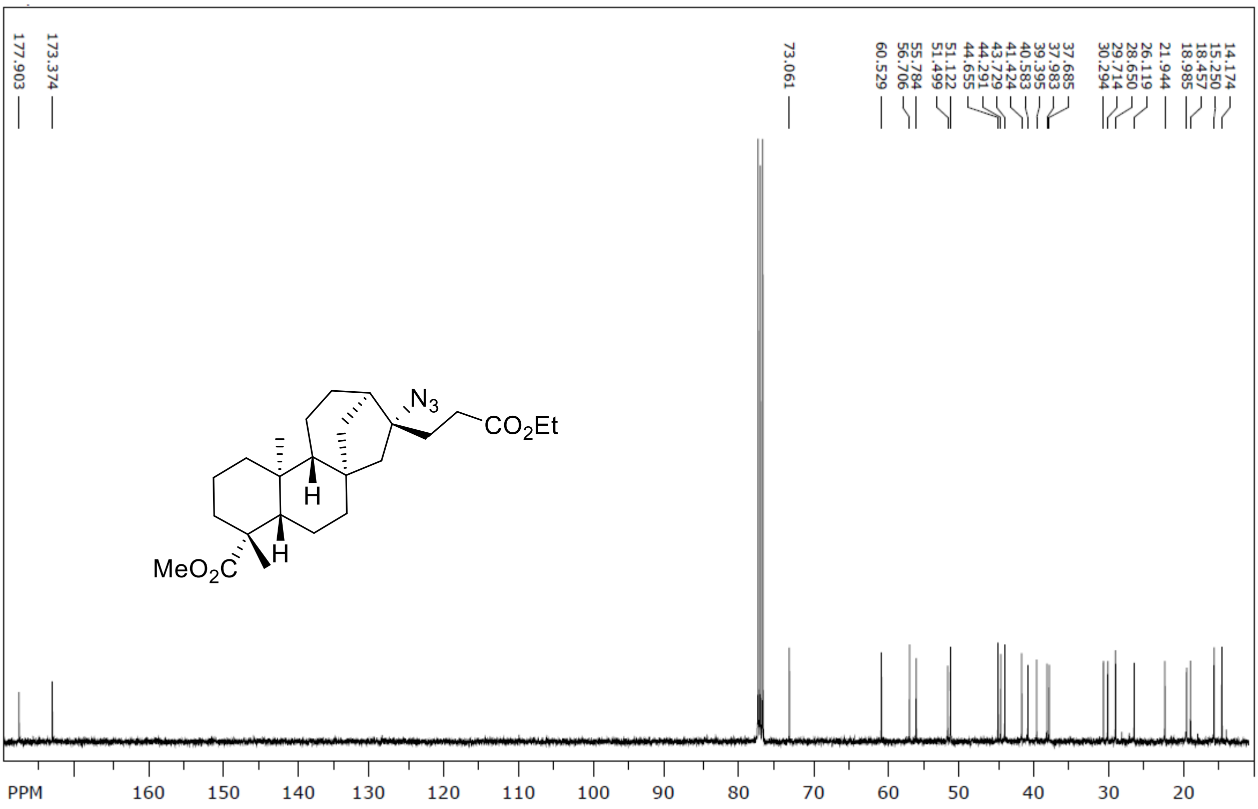

^{13}C NMR spectrum of azide **11**.

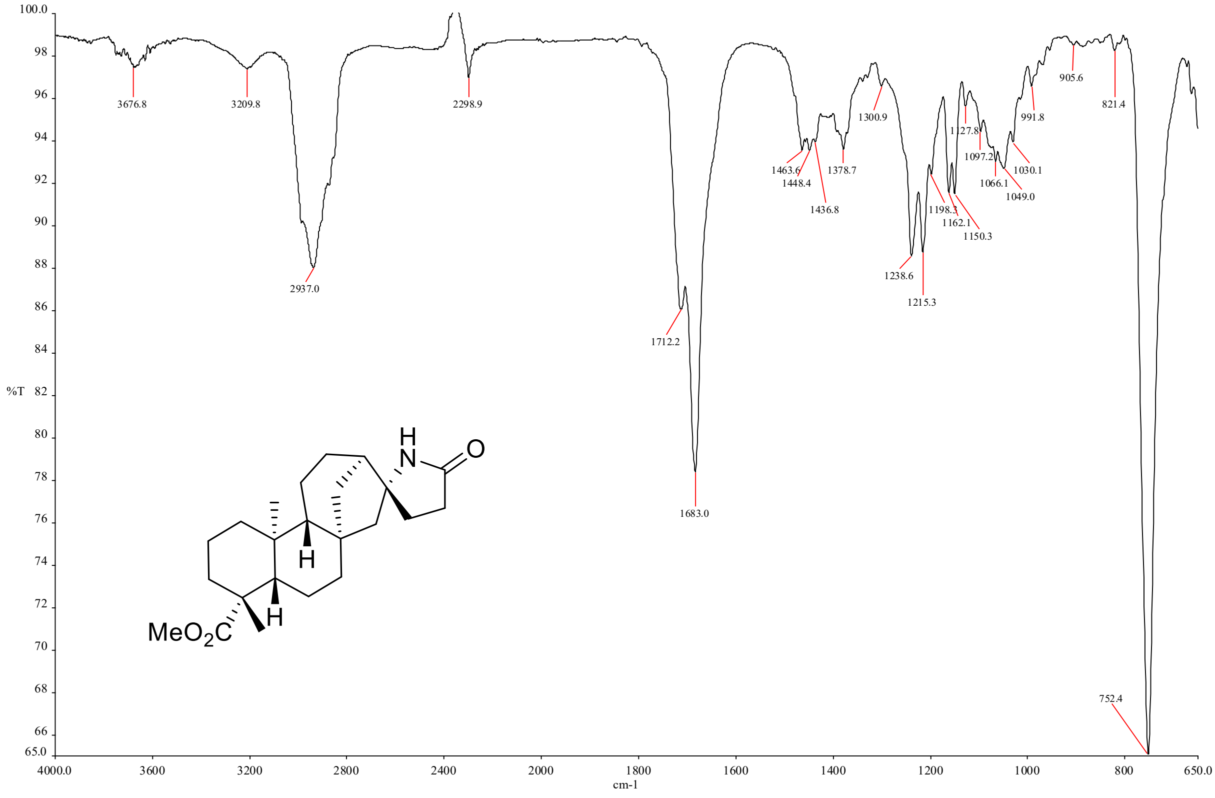

IR spectrum of lactam **12**.

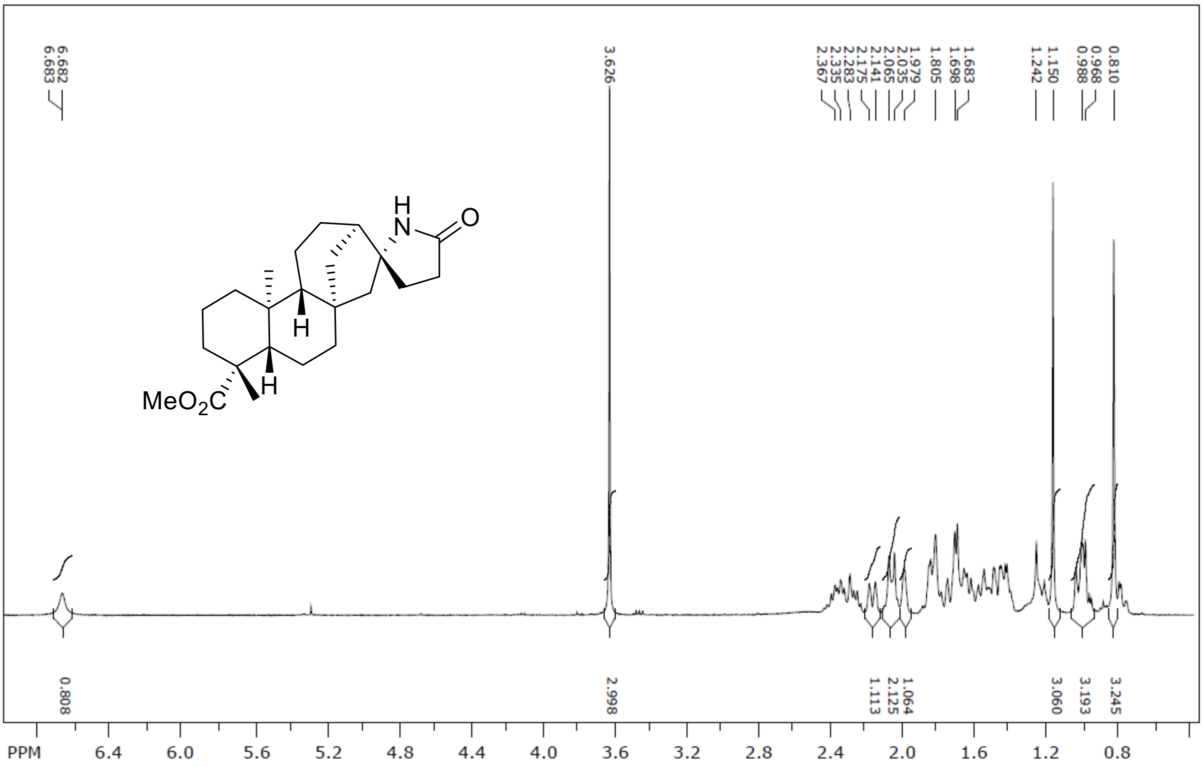

^{1}H NMR spectrum of lactam **12**.

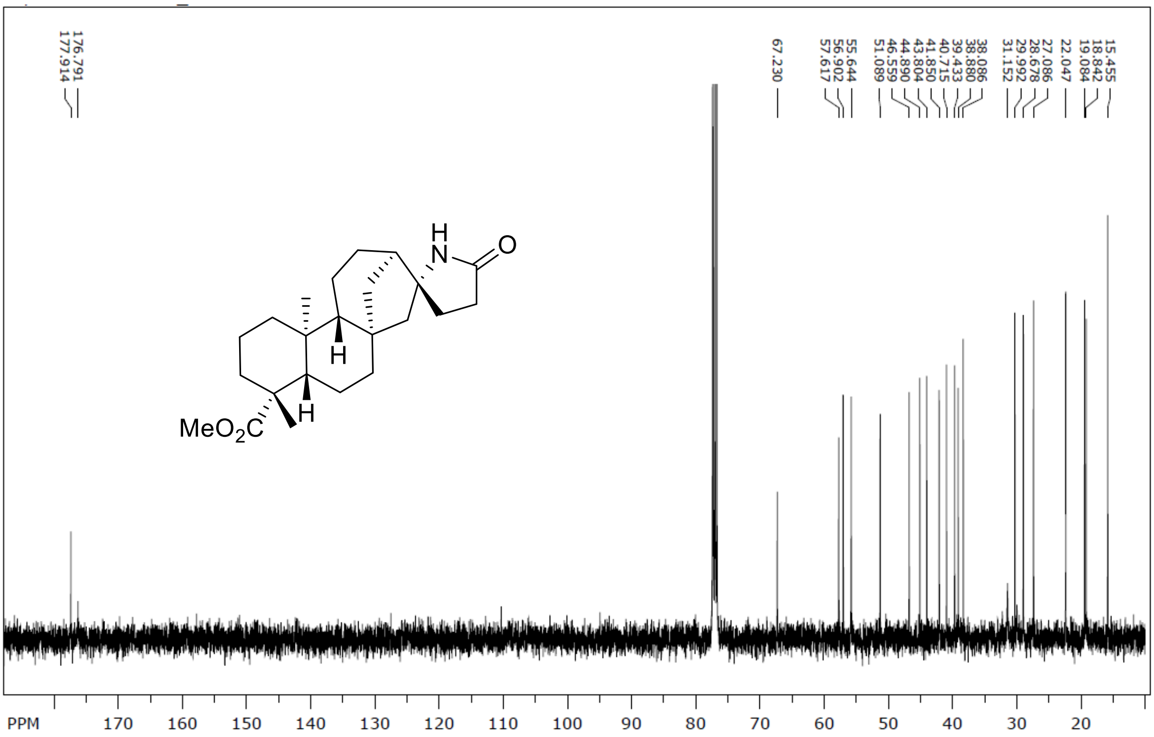

^{13}C NMR spectrum of lactam **12**.

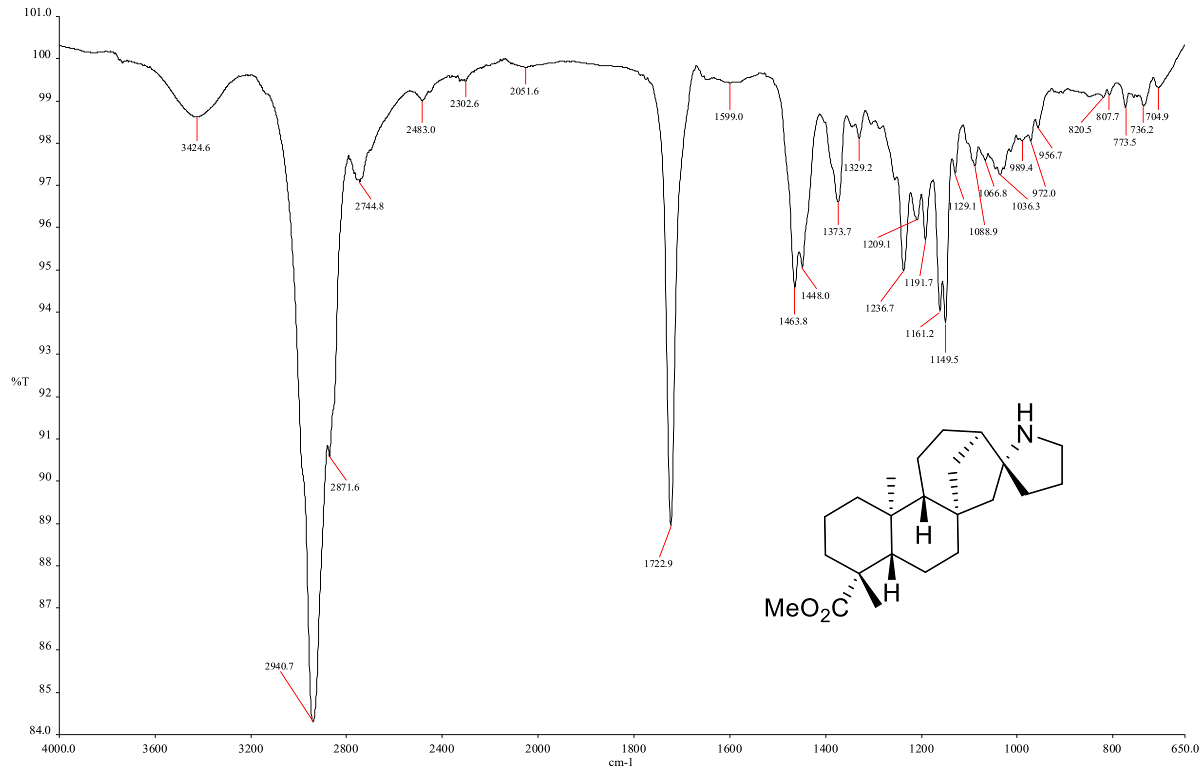

IR spectrum of pyrrolidine **13**.

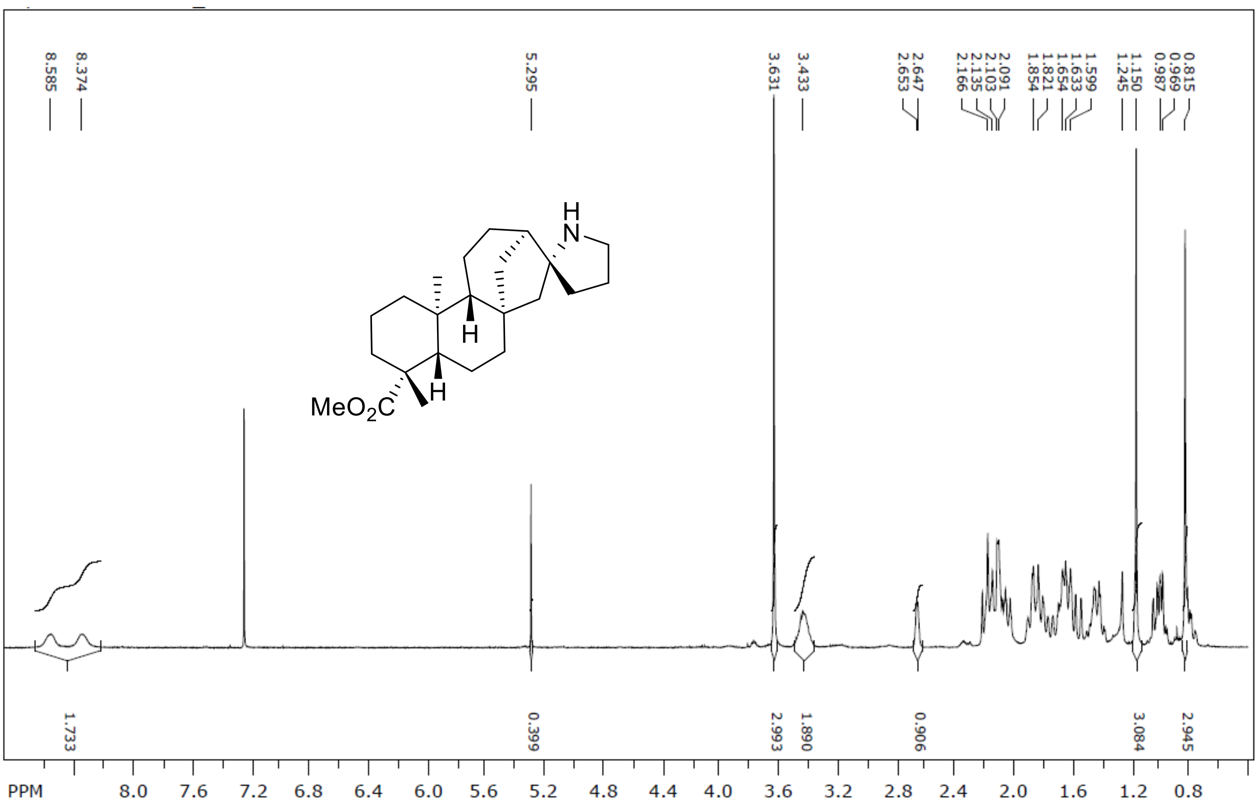

^{1}H NMR spectrum of pyrrolidine **13**.

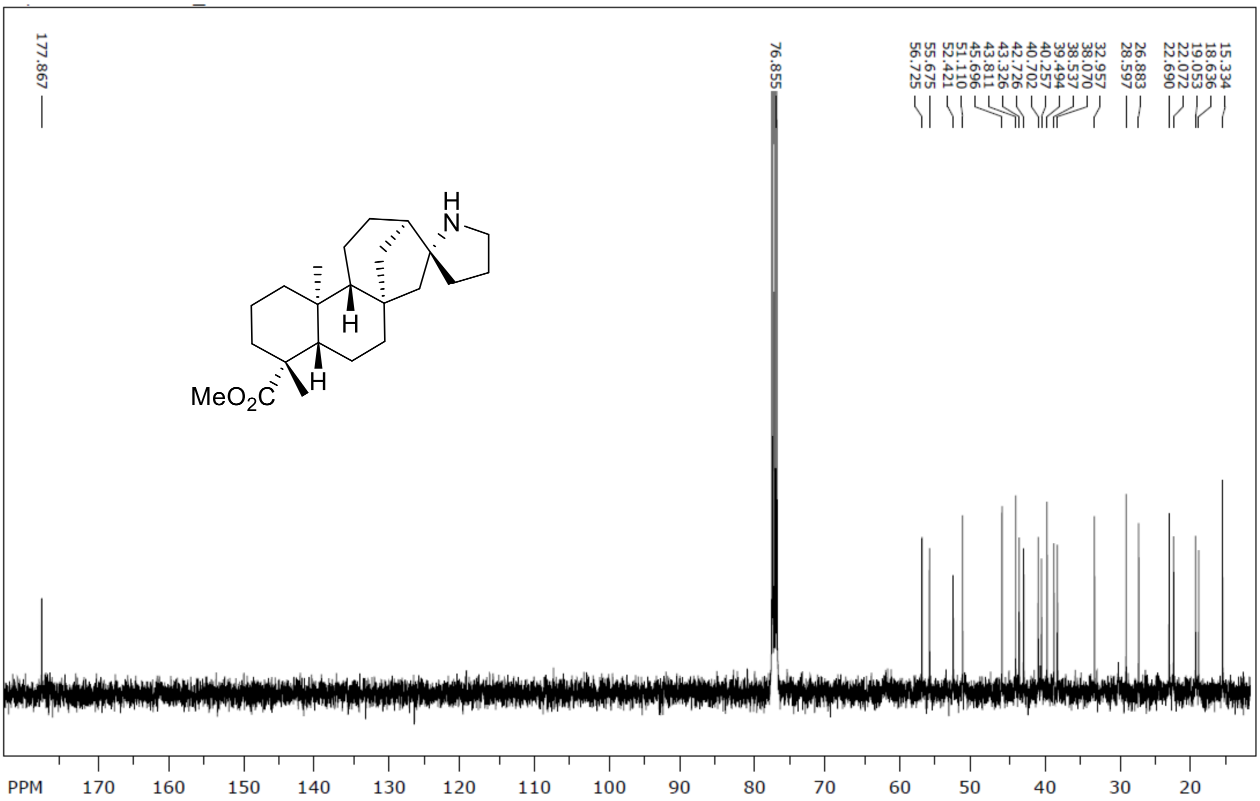

^{13}C NMR spectrum of pyrrolidine **13**.

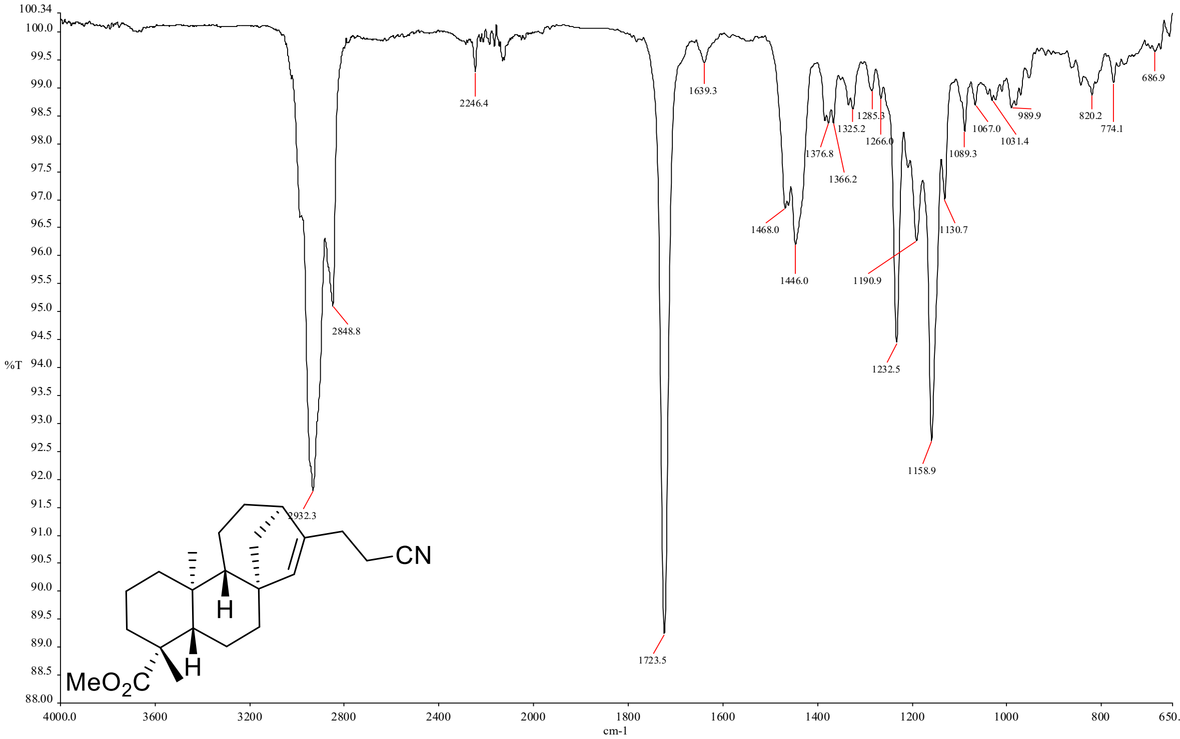

IR spectrum of nitrile **16**.

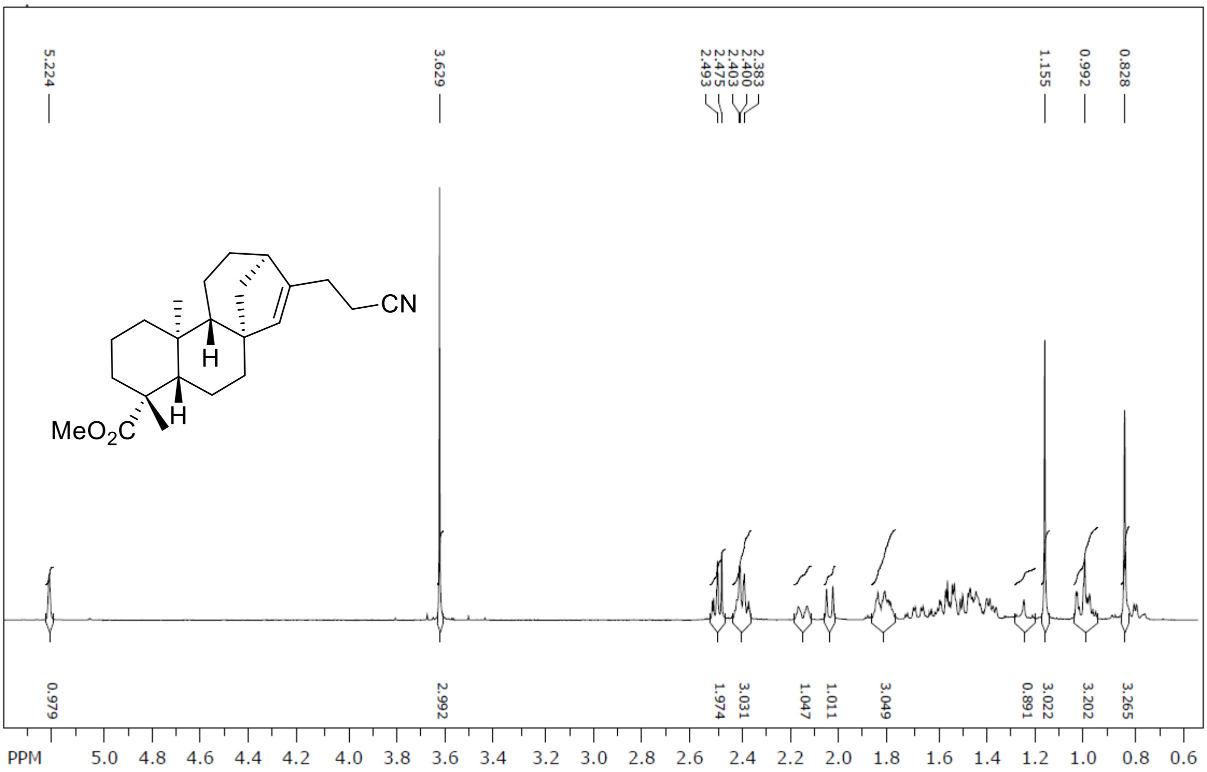

^{1}H NMR spectrum of nitrile **16**.

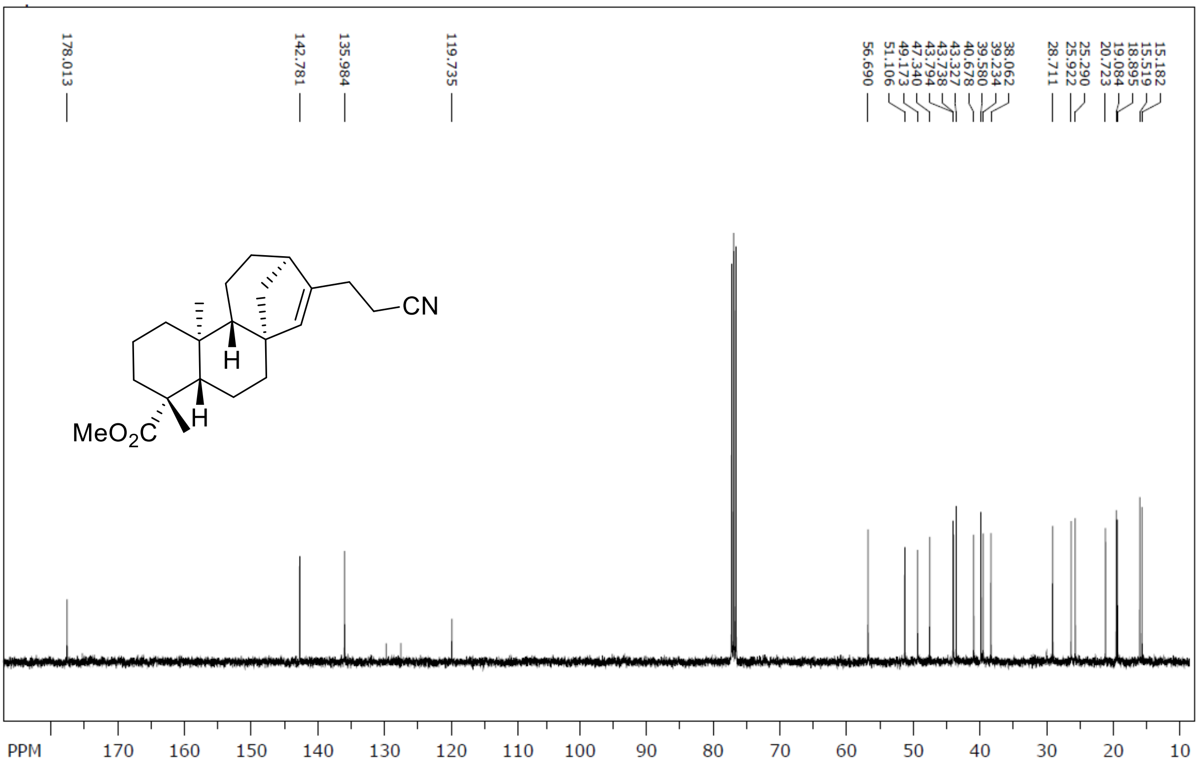

^{13}C NMR spectrum of nitrile **16**.

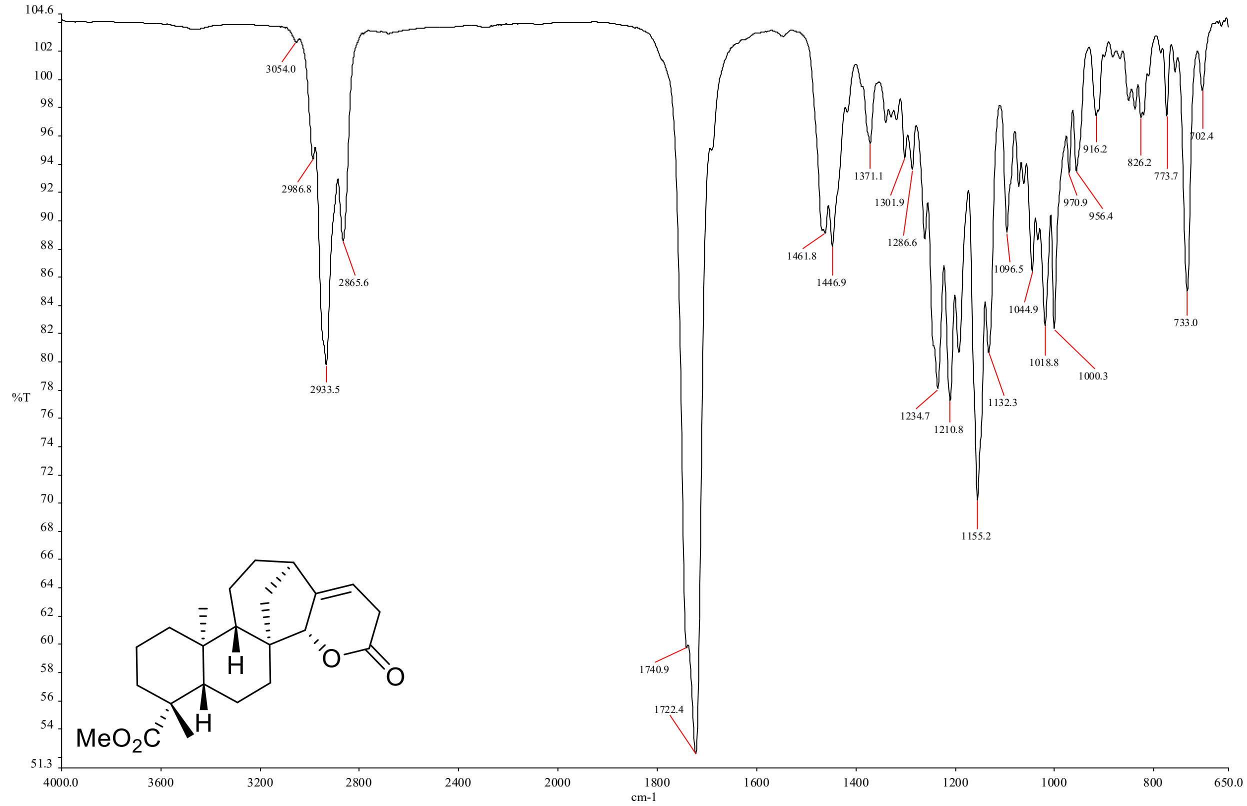

IR spectrum of lactone **22**.

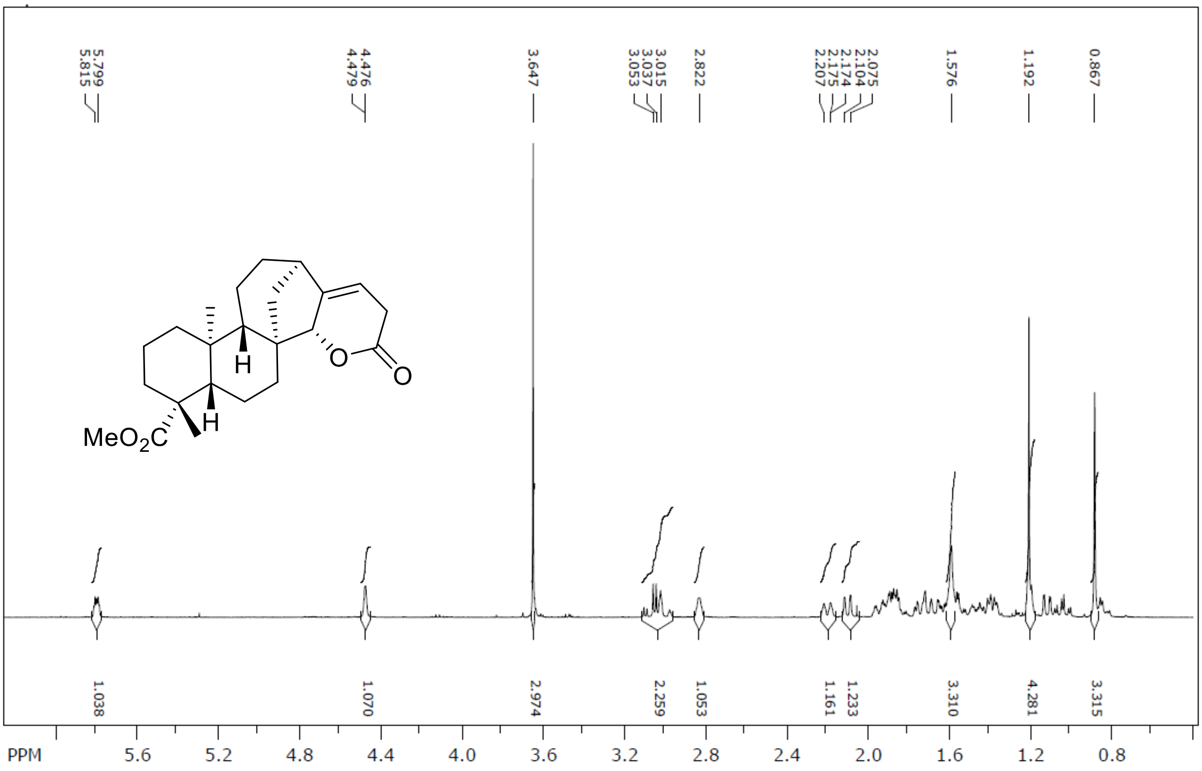

^{1}H NMR spectrum of lactone **22**.

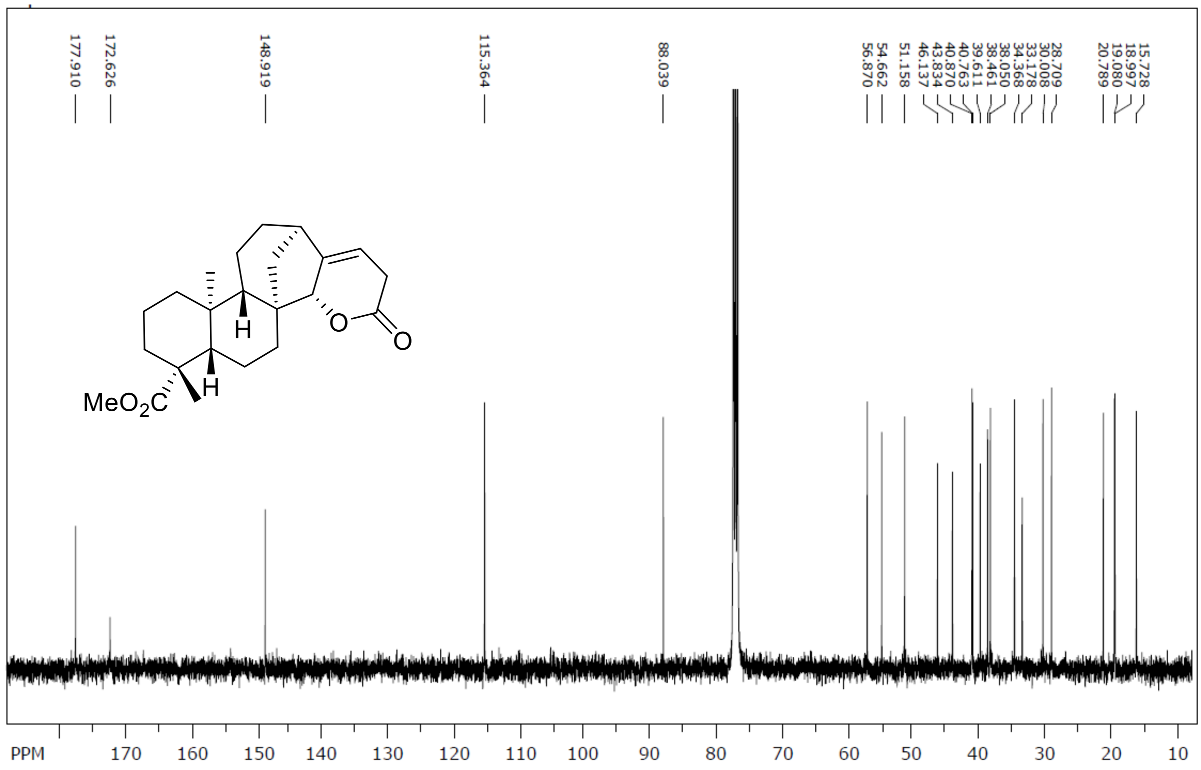

^{13}C NMR spectrum of lactone **22**.

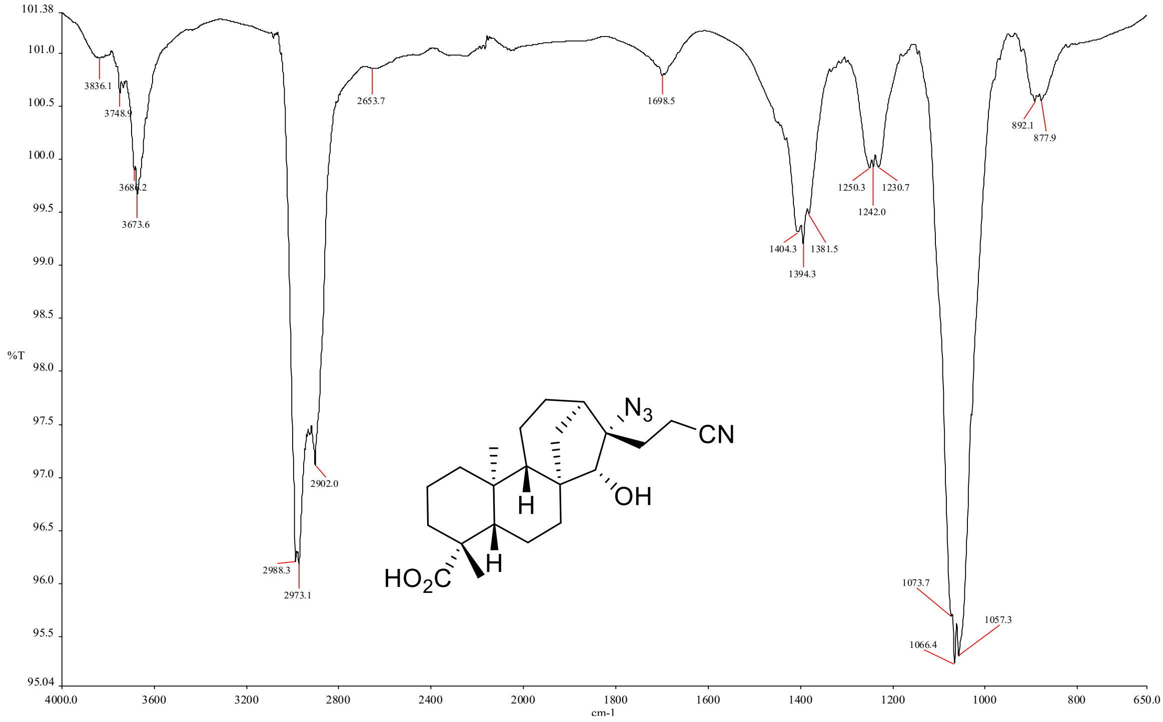

IR spectrum of azide **25**.

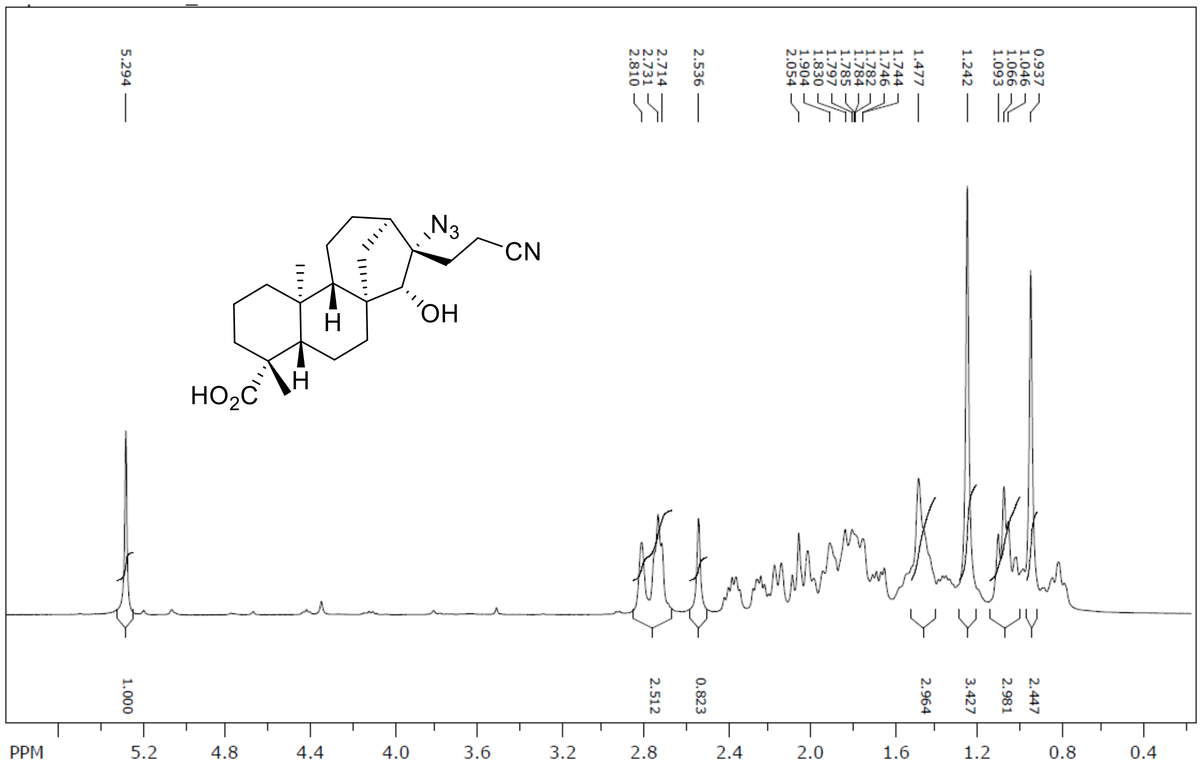

^1H NMR spectrum of azide **25**.

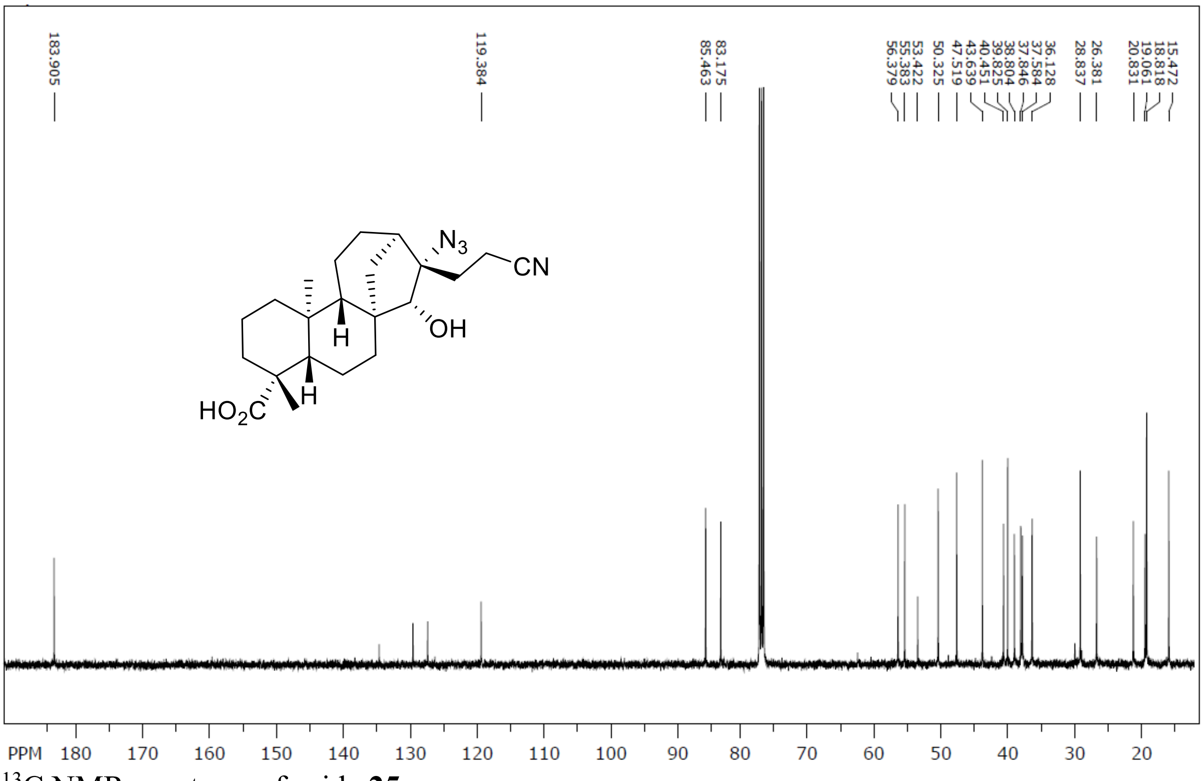

^{13}C NMR spectrum of azide **25**.

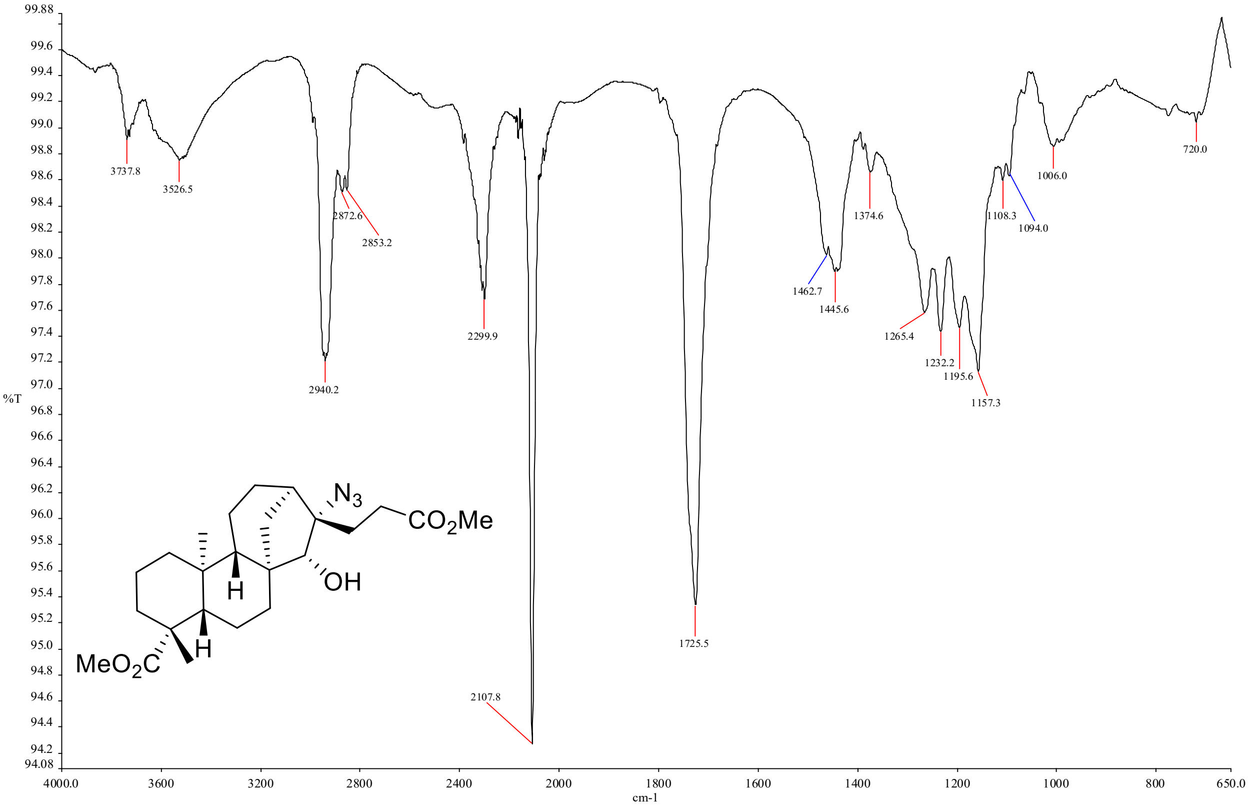

IR spectrum of azide **26**.

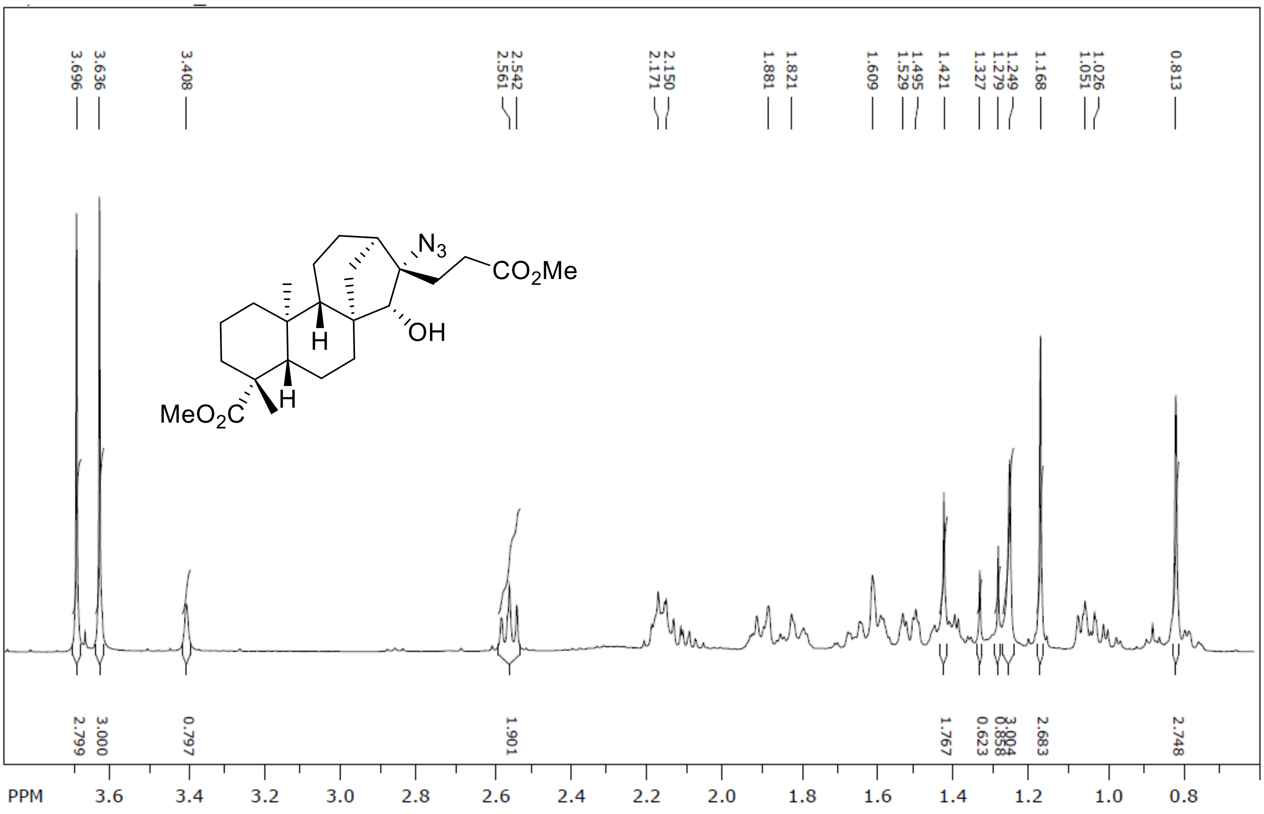

^{1}H NMR spectrum of azide **26**.

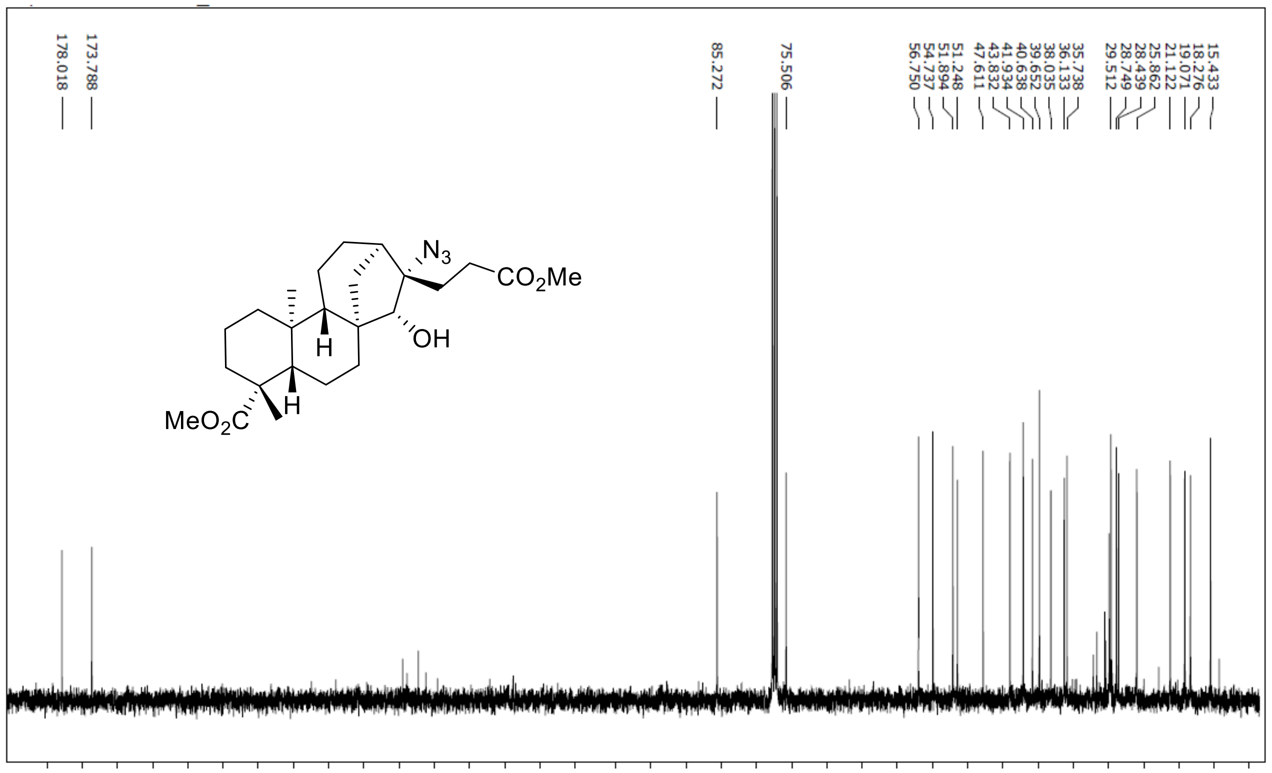

^{13}C NMR spectrum of azide **26**.

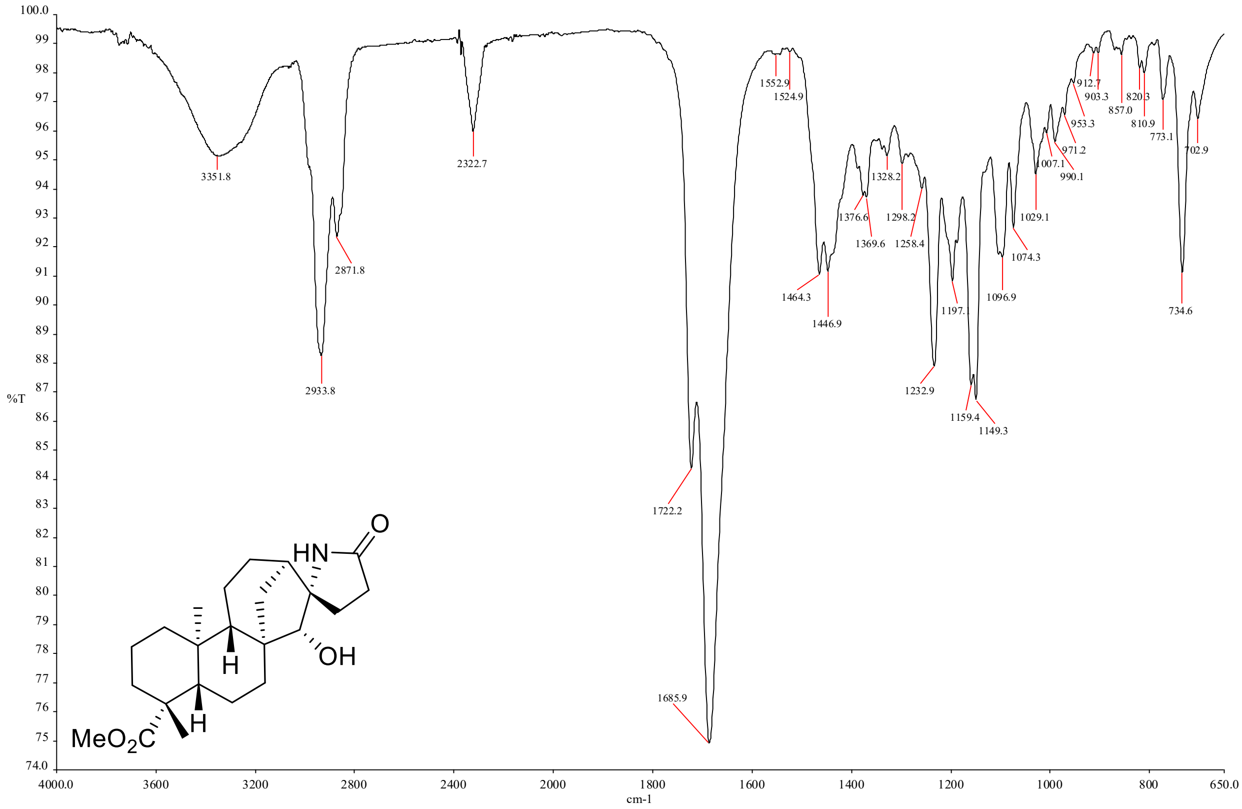

IR spectrum of lactam **27**.

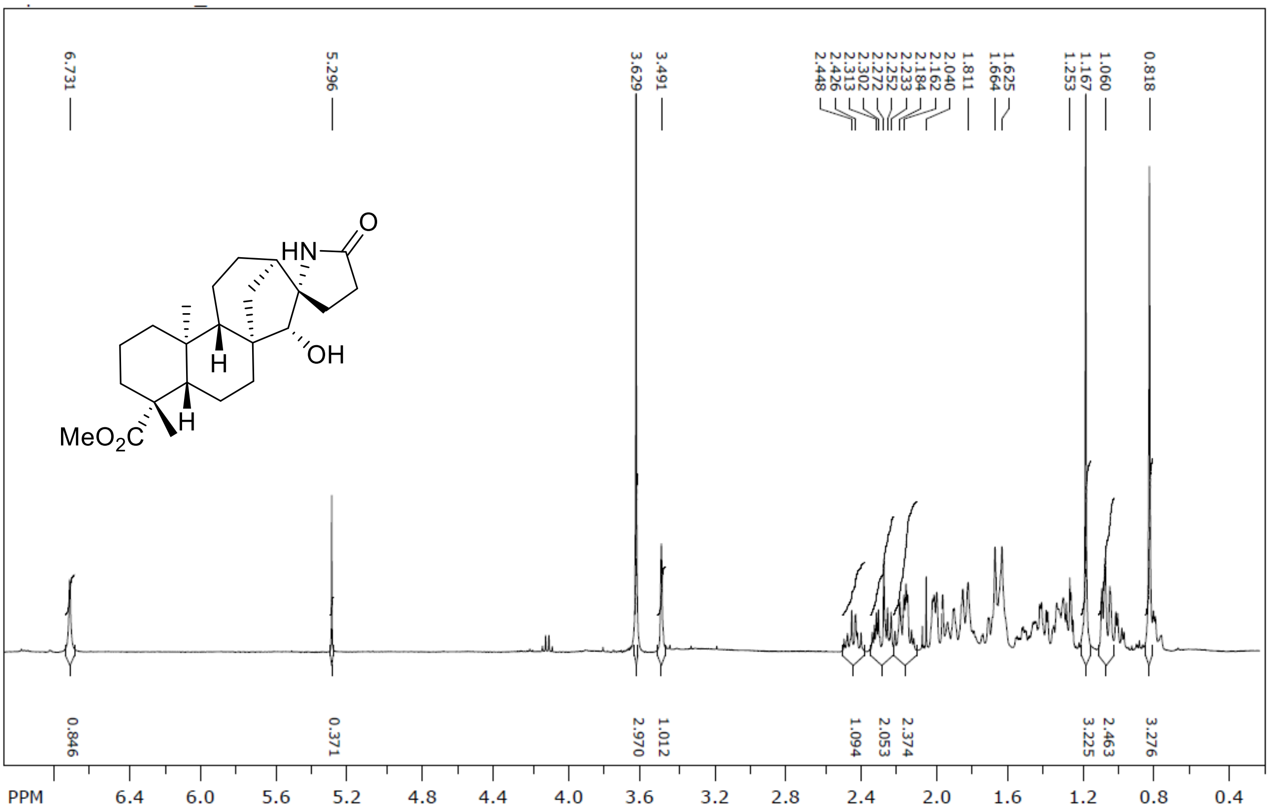

^{1}H NMR spectrum of lactam **27**.

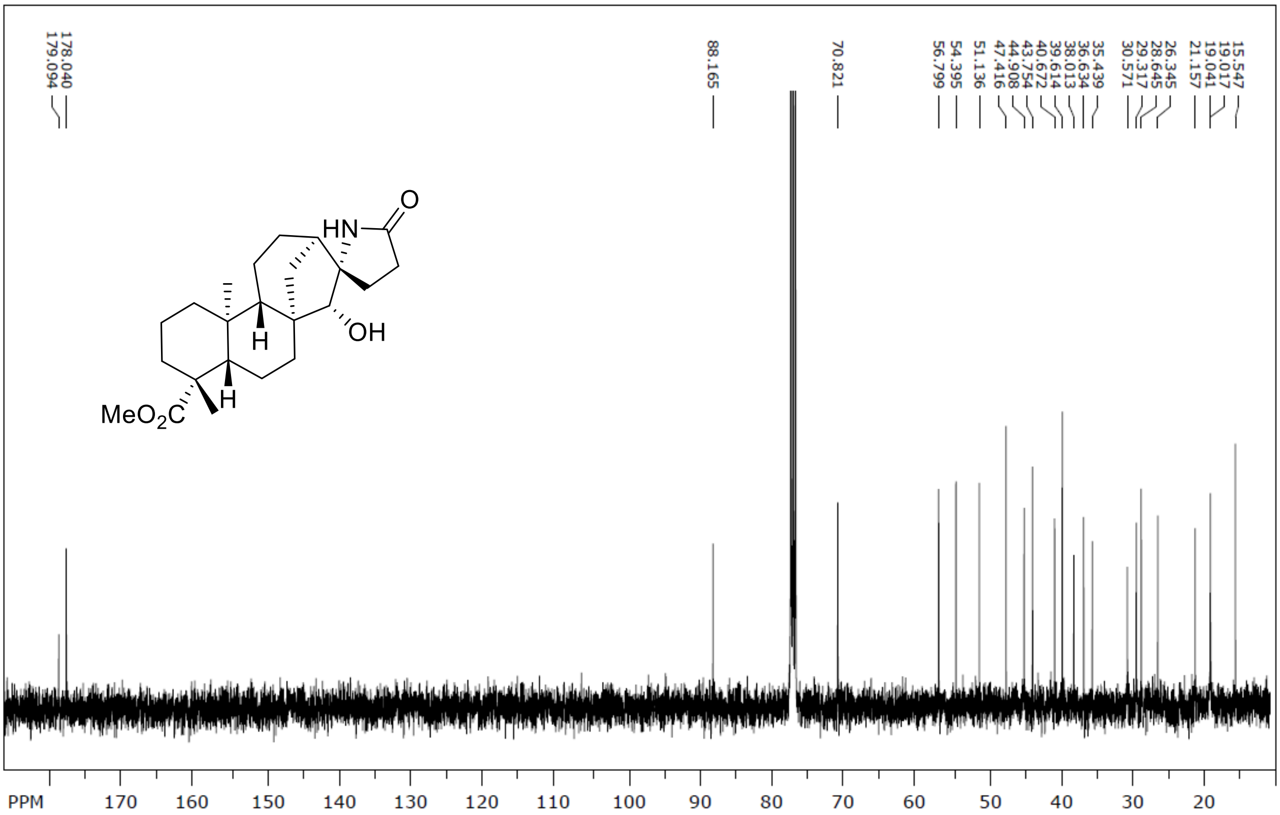

^{13}C NMR spectrum of lactam **27**.

Table S14. Antiproliferative activity (IC_{50}) and selectivity indexes (SI)* for selected *ent*-kauranic derivatives. **DT** – Docetaxel; **SP** – Staurosporine.

**Compound**	**Descriptor**	**IC_{50}, µM / SI**							
		**hTERT RPE-1**	**Capan-1**	**HCT-116**	**NCI-H460**	**DND-41**	**HL-60**	**K-562**	**Z-138**
**7**	IC_{50}	29.2±1.2	37.1±3.9	30.7±16.4	12.0±1.5	36.5±0.7	19.6±7.7	24.7±11.8	27.6±15.5
	SI		-	-	2.4	-	1.5	1.2	1.1
**11**	IC_{50}	1.8±0.4	1.7±0.5	1.0±0.4	1.3±0.1	1.4±0.3	3.0±1.4	8.8±2.2	0.5±0.2
	SI		1.1	1.8	1.4	1.3	-	-	3.6
**12**	IC_{50}	7.6±0.5	1.2±0.7	0.8±0.2	1.4±0.1	21.4±10.1	38.3±17.8	36.4±11.5	52.0±33.4
	SI		6.3	9.5	5.4	-	-	-	-
**13**	IC_{50}	32.2±2.0	11.5±1.0	11.6±0.5	1.0±0.1	6.1±1.1	8.3±1.3	3.2±1.0	9.5±0.9
	SI		2.8	2.8	32.2	5.3	3.9	10.1	3.4
**14**	IC_{50}	26.2±0.9	44.5±8.3	5.8±0.1	31.1±1.6	44.5±4.8	46.2±9.1	41.0	22.7
	SI		-	4.5	-	-	-	-	1.2
**16**	IC_{50}	38.2±5.1	18.1±3.4	19.0±5.7	15.4±6.0	7.8±1.0	33.6±2.6	58.4±2.5	22.1±11.4
	SI		2.1	2.0	2.5	4.9	1.1	-	1.7
**22**	IC_{50}	1.4±0.1	1.3±0.6	0.5±0.2	1.4±0.3	2.3±0.3	3.7±1.3	2.5±0.1	2.4±1.1
	SI		1.1	2.8	1	-	-	-	-
**24**	IC_{50}	31.7±3.1	33.2±7.6	18.8±8.0	41.8±5.3	38.9±4.1	49.3±5.0	76.3±1.4	25.2±0.3
	SI		-	1.7	-	-	-	-	1.3
**25**	IC_{50}	33.6±4.5	6.4±1.5	34.4±0.5	34.0±2.9	36.4±9.8	>100	>100	52.0±17.4
	SI		5.3	-	-	-	-	-	-
**26**	IC_{50}	25.9±0.3	8.3±1.1	9.9±2.7	0.6±0.2	6.6±1.3	41.1±15.9	36.9±0.2	21.6±5.4
	SI		3.1	2.6	43.2	3.9	-	-	1.2
**27**	IC_{50}	27.7±4.3	3.7±1.7	4.0±3.7	1.9±0.2	8.3±2.0	38.0±11.8	56.5±2.8	39.8±10.9
	SI		7.5	6.9	14.6	3.3	-	-	-
**DT**	IC_{50}, nM	18.7±4.8	4.2±1.8	2.2±0.8	5.5±1.3	4.7±1.2	4.3±1.6	5.2±1.2	3.7±0.7
**SP**	IC_{50}, nM	1.0	6.2±1.8	1.5	2.2±0.8	8.6±1.5	9.1±1.6	27.9±3.2	6.7±4.4

Tumor cell lines : Capan-1 (pancreatic adenocarcinoma), HCT-116 (colorectal carcinoma), NCI-H460 (lung carcinoma), DND-41 (acute lymphoblastic leukemia), HL-60 (acute myeloid leukemia), K-562 (chronic myeloid leukemia), and Z-138 (non-Hodgkin lymphoma).

*Values of SI are calculated only for compounds that exhibit lower IC_{50} values towards tumor cell lines than hTERT RPE-1 normal cells.

REFERENCES

1. *CrysAlisPro (Version 1.171.34.44)*; Oxford Diffraction Ltd.: Yarnton, Oxfordshire, UK, 2010;
2. Macchi, P.; Bürgi, H.-B.; Chimpri, A.S.; Hauser, J.; Gál, Z. Low-Energy Contamination of Mo Microsource X-Ray Radiation: Analysis and Solution of the Problem. *J. Appl. Crystallogr.* **2011**, *44*, 763–771, doi:10.1107/S0021889811016232.
3. Sheldrick, G.M. *SHELXT* – Integrated Space-Group and Crystal-Structure Determination. *Acta Crystallogr. Sect. Found. Adv.* **2015**, *71*, 3–8, doi:10.1107/S2053273314026370.
4. Sheldrick, G.M. Crystal Structure Refinement with *SHELXL*. *Acta Crystallogr. Sect. C Struct. Chem.* **2015**, *71*, 3–8, doi:10.1107/S2053229614024218.
5. Dolomanov, O.V.; Bourhis, L.J.; Gildea, R.J.; Howard, J.A.K.; Puschmann, H. *OLEX2* : A Complete Structure Solution, Refinement and Analysis Program. *J. Appl. Crystallogr.* **2009**, *42*, 339–341, doi:10.1107/S0021889808042726.
